# Roughness perception: A multisensory/crossmodal perspective

**DOI:** 10.3758/s13414-022-02550-y

**Published:** 2022-08-26

**Authors:** Nicola Di Stefano, Charles Spence

**Affiliations:** 1 National Research Council, Institute for Cognitive Sciences and Technologies, Rome, Italy; 2 University of Oxford, Oxford, UK

**Keywords:** Auditory consonance/dissonance, Human vocalizations, Astringency, Intersensory, Taste, Touch, Olfaction, Vision

## Abstract

Roughness is a perceptual attribute typically associated with certain stimuli that are presented in one of the spatial senses. In auditory research, the term is typically used to describe the harsh effects that are induced by particular sound qualities (i.e., dissonance) and human/animal vocalizations (e.g., screams, distress cries). In the tactile domain, roughness is a crucial factor determining the perceptual features of a surface. The same feature can also be ascertained visually, by means of the extraction of pattern features that determine the haptic quality of surfaces, such as grain size and density. By contrast, the term *roughness* has rarely been applied to the description of those stimuli perceived via the chemical senses. In this review, we take a critical look at the putative meaning(s) of the term *roughness*, when used in both unisensory and multisensory contexts, in an attempt to answer two key questions: (1) Is the use of the term ‘roughness’ the same in each modality when considered individually? and (2) Do crossmodal correspondences involving roughness match distinct perceptual features or (at least on certain occasions) do they merely pick-up on an amodal property? We start by examining the use of the term in the auditory domain. Next, we summarize the ways in which the term *roughness* has been used in the literature on tactile and visual perception, and in the domain of olfaction and gustation. Then, we move on to the crossmodal context, reviewing the literature on the perception of roughness in the audiovisual, audiotactile, and auditory-gustatory/olfactory domains. Finally, we highlight some limitations of the reviewed literature and we outline a number of key directions for future empirical research in roughness perception.

## Introduction

More than two millennia ago, Aristotle conceived of roughness as one of the putative stimulus properties that could be apprehended through different senses: “For the perception of magnitude, figure, roughness, smoothness, and sharpness and bluntness, in solid bodies, is the common function of all the senses, and if not all, then at least the common function of sight and touch” (Aristotle, 1906, p. 442b). Aristotle went on to suggest that ‘common sensibles’ are those features of the world, such as roughness, that can be perceived in their own right by different senses (Aristotle, 1907, pp. 418a10–11, 19). As observed by Johnstone (2021), this leaves it somewhat unclear as to whether these common sensibles should be common to all of the senses or just to two or more of them. Aligning with most commentators (e.g., Knuuttila, 2008), Johnstone takes Aristotle’s considered view to have been that common sensibles are perceptible in their own right by more than one sense but need not necessarily be perceptible by all five of the commonly accepted senses. In particular, with respect to roughness, Aristotle seems to conceive of it primarily as opposed to smoothness in the domain of touch, while he did not assign a prominent role to auditory roughness.

Interestingly, in the 19th century, the eminent German psychophysicist Hermann von Helmholtz considered roughness to be a crucial concept in the explanation of one of the most fundamental distinctions in the perception of sounds—namely, the distinction between consonance and dissonance (Helmholtz, 1954). Additionally, recent multidisciplinary findings have also demonstrated that roughness constitutes a crucial component in human and animal communication. Studies have shown that the roughness of vocalization is typically associated with distress or danger, such as screaming (Arnal et al., 2015), roaring (Kleisner et al., 2021; Raine et al., 2019), crying (Koutseff et al., 2018; Young et al., 2016), thus capturing the attention of the perceiver and evoking aversive responses (e.g., Anikin et al., 2021; for animals, see Götz & Janik, 2010; Hechavarría et al., 2020; Marx et al., 2021; Soltis et al., 2011). Biological reasons to explain this evidence point to the relationship between the physical structure of sound signals and the motivation underlying their use, with harsh and rough sounds being used in hostile and aggressive encounters with other animals (Morton, 1977). Such a biological link likely impacts on the aesthetic effect of rough sounds in humans, with the latter typically being perceived as unpleasant, or less pleasant, than nonrough (or less rough) sounds (Helmholtz, 1954; Zwicker & Fastl, 2006).

The theorizations by Aristotle and Helmholtz are representative of the way in which roughness has typically been conceptualized in the West (i.e., as a sensory feature, one that has primarily been associated with auditory and tactile stimuli). The latter are typically related to perceptual objects (e.g., surfaces) that can also be inspected visually. In this sense, it would appear that both visual and tactile perception can pick up on the same stimulus properties, namely surface roughness, that is typically perceived as unpleasant, at least when experienced haptically (Ackerley et al., 2014; Ekman et al., 1965; Essick et al., 2010; Kitada et al., 2012; Verrillo et al., 1999), presumably because rough surfaces are more likely to damage one’s skin than smooth surfaces. Much less frequently, and only far more recently, has roughness been caused by/attributed to food stimuli perceived through the chemical senses^1^ and, in some cases, it is used metaphorically to identify other gustatory properties, such as, for example, astringency, or to refer to a change in the perception of the sensing surface, namely the tongue (Green, 1993; Lee & Lawless, 1991; Corrigan Thomas & Lawless, 1995).

This review addresses the general question of whether roughness can be experienced multisensorially (or influenced crossmodally), or whether instead it is only ever experienced within individual senses (e.g., audition and touch) and thus metaphorically used in relation to other senses (e.g., gustation). Relatedly, we will also try to distinguish between crossmodal perception—that is, when the roughness of a stimulus perceived through one sense, for example, hearing (a sound), is shown to affect the roughness of a stimulus perceived through a different sense, for example, touch (fabrics)—and multisensory perception (or integration)—that is, when different sensory data inform an individual about the same perceptual object, for example, one both feels and sees the same surface. To do this, we take a critical look at the putative meaning(s) of the term *roughness* when used in both a unisensory and multisensory context. We start by investigating the use of the term *roughness* in the auditory and tactile modalities, as these are the two most prototypical contexts in which the term has ordinarily been used. As far as hearing is concerned, we will focus especially on the acoustic property of roughness, both of sounds and vocalizations, rather than considering the sounds produced by the touching of rough surfaces (note that the latter meaning is most frequent in the literature on the perception of surface texture). Next, we summarize the various ways in which the term *roughness* has been used in the literature on visual perception and thereafter in the literature on the chemical senses. We move on to look at the crossmodal context, reviewing the literature on the perception of roughness in the audiovisual, audiotactile, and auditory-gustatory/olfactory domains. Finally, we highlight some limitations of the reviewed literature and we outline a number of key directions for future empirical research in roughness perception.

As will become clear, there are several key questions at stake in this review. As we try to address the fundamental question of whether the use of the term ‘roughness’ is the same in each modality when considered individually, the following related questions will also be addressed: (1) Is roughness primarily experienced in (a) certain sensory domain(s) (e.g., tactile/auditory), and only metaphorically referred to in other senses? If so, across which particular senses, does it apply? (2) What are the different possible sensory components of roughness? Are different components of roughness present for some senses, or crossmodal combinations, but not other sensory domains? (3) Does roughness elicit negative sensory experience in the perceiver no matter which sense the term refers to? (4) Finally, can the different kinds of roughness affect each other crossmodally or else be integrated multisensorially?

## Auditory roughness

### Musical tones: Roughness and dissonance

Roughness has been defined as the auditory effect elicited by “beating at frequencies in the range 20–300 Hz” (Parncutt, 1989, p. 178; 15–300 Hz according to Zwicker & Fastl, 2006, p. 257), reaching a maximum at a frequency of around 70 Hz (Parncutt, 1989, p. 58; Zwicker & Fastl, 2006, p. 257).^2^ In his groundbreaking treatise on acoustics and music perception, *On the Sensations of Tone*, Helmholtz (1954) introduced roughness as a key concept in the explanation of musical dissonance. In general, the term dissonance, and its opposite—namely, consonance, refer to the different effects generated on the listener by two tones played either simultaneously or else one after the other (i.e., sequentially), with dissonances typically being perceived as unstable and negatively valenced; by contrast, consonances are typically perceived as smooth, harmonious, and positively valenced (Harrison & Pearce, 2020; Malmberg, 1918; Maslennikova et al., 2013; Passynkova et al., 2007). Based on the physical laws that govern the combination of sounds, Helmholtz claimed that the different effects that are generated by consonance and dissonance are essentially linked to roughness. In particular, he assumed roughness to be “the peculiar character of dissonance,” while the absence of roughness characterizes consonance, which Helmholtz considered to be an “exceptional case” (Helmholtz, 1954, p. 194).

For Helmholtz, roughness was related to the phenomenon of beating, which occurs when the harmonics of two tones sounding simultaneously are not spaced sufficiently far apart and therefore mutually interfere, giving rise to a modified waveform with a rhythmic oscillation in vibration pattern or amplitude. For a complex-tone pair with given structures of partial amplitudes, the number of beating partials is smallest when the fundamental frequencies are related by a ratio of small integers (Terhardt, 1974). For example, an interval of octave (2:1) has all the harmonics that coincide, thus resulting in the absence of beating and minimal roughness (i.e., maximal consonance). On the other hand, an octave slightly mistuned by a ε factor (f_2_ = 2f_1_ + ε), will generate beating at the frequency of ε (see Plomp & Levelt, 1965).

Helmholtz attempted to explain the link between beating and roughness in physiological terms: “The nerves of hearing feel these rapid beats as *rough* and unpleasant, because every intermittent excitement of any nervous apparatus affects us more powerfully than one that lasts unaltered. With this there is possibly associated a psychological cause. The individual pulses of tone in a dissonant combination give us certainly the same impression of separate pulses as slow beats, although we are unable to recognize them separately and count them; hence they form a *tangled* mass of tone, which cannot be analyzed into its constituents. The cause of the unpleasantness of dissonance we attribute to this *roughness* and *entanglement*. The meaning of this distinction may thus be briefly stated: *Consonance is a continuous, dissonance an intermittent sensation of tone*. . . . Consonance is the blending of a higher with a lower tone. Dissonance is incapacity to mix, when two tones cannot blend, but appear rough to the ear” (Helmholtz, 1954, p. 226).

**Fig. 1:**
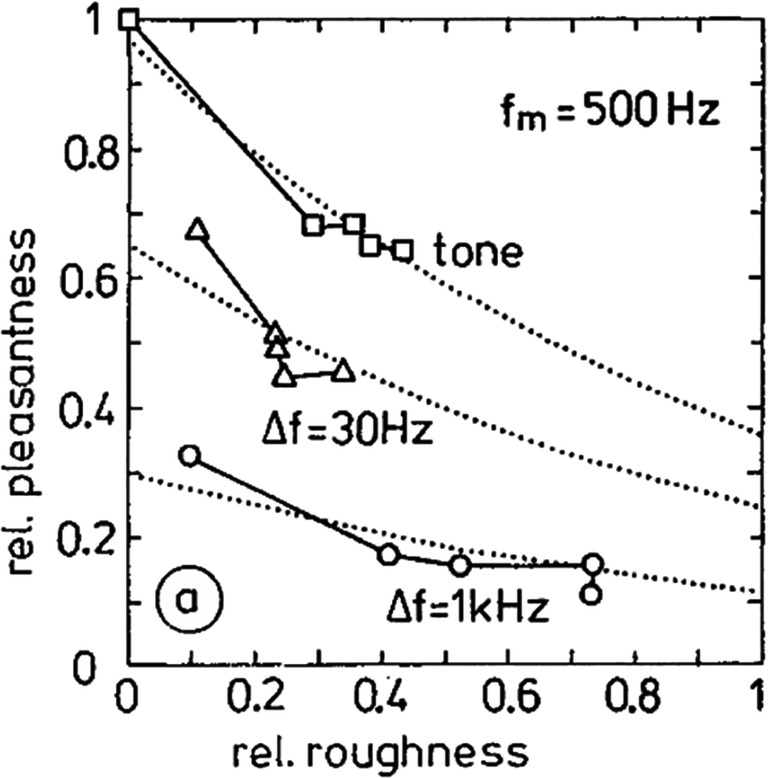
Relative (rel.) pleasantness as a function of relative (rel.) roughness with bandwidth as the parameter (Reprinted from Zwicker & Fastl, 2006, p. 244)

For a given frequency, the smallest band of frequencies that evoke beating is called the “critical bandwidth.” Helmholtz claimed that the critical bandwidth is rooted in the biological properties of the inner ear (i.e., in the mechanics of the basilar membrane). In fact, according to the tonotopic theory, each site along the basilar membrane is characterized by a resonance frequency, based on the morphological characteristics of the organic tissue, such as thickness, elasticity, and rigidity (Helmholtz, 1954, pp. 138–141). Different frequencies trigger different modes of membrane vibration (i.e., with different points of maximum oscillation). When two sounds within a critical bandwidth hit the ear, the membrane is unable to analyze the auditory information, and portions of the membrane that overlap with each other start vibrating, giving rise to the phenomenon of beats and inducing sensory roughness in the perceiver. It is thus not surprising that roughness has been considered among the factors that affect sensory pleasantness, together with others elementary auditory sensations such as sharpness, tonality, and loudness (Zwicker & Fastl, 2006, p. 243). In particular, the increase of roughness is associated with decreased pleasantness (see Fig. 1). In addition to models of roughness based on the spectral composition of energy falling within critical bands (Hutchinson & Knopoff, 1978), different approaches focused on the influence of temporal parameters by means of amplitude-modulated stimuli, have demonstrated that roughness depends on the frequency and depth of the modulation (Fastl, 1977; Mathes & Miller, 1947; Terhardt, 1974; see also Pressnitzer & McAdams, 1999, for the effects of phase on roughness perception).

From Helmholtz to the present day, roughness has always been considered crucial to any explanation of consonance and dissonance (e.g., Eerola & Lahdelma, 2022). Recently, Parncutt et al. (2019) analyzed a large database of vocal polyphony from 13th-to-19th-century Western classical music to classify the most frequent trichords. They then explored consonance and dissonance concepts comparing the prevalence of chord types in different periods with predictions based on quantitative models. Each model was based on a different explanatory factor of consonance—namely, diatonicity, roughness, familiarity, unevenness, and harmonicity. Parncutt and colleagues demonstrated that roughness, together with harmonicity and familiarity, contributed most to determine the history of consonance and dissonance in Western music. Interestingly, cross-cultural studies have shown that the perceptual attribute of roughness is also salient to non-Western listeners. Trying to assess whether the perception of consonance is universal, McDermott et al. (2016) exposed a group of Tsimane’ from Bolivia, a population naïve to Western music, to a wide variety of stimuli that varied in terms of roughness, among other auditory features. Their results suggested that, also for those listeners, roughness is a salient and negatively valenced feature of auditory stimuli. Taken together, therefore, these studies indicate that roughness is one of the most salient acoustic features. It appears to be correlated with consonance/dissonance, independent of musical system, and is typically associated with a negative aesthetic judgement by the listener.

### Auditory roughness in human and animal vocalization and communication

Roughness plays a prominent role in determining the perceived quality of auditory stimuli as compared with other acoustic features, such as loudness (Anikin et al., 2020; Anikin et al., 2021; Zhao et al., 2019). Several biological reasons have been put forward to provide support for this claim, indicating a relationship between the physical structure of sound signals and the motivation underlying their use. For example, as suggested by Morton (1977), birds and mammals use harsh and rough sounds when hostile and in “face to face” aggressive encounters with other animals. On the other hand, they tend to produce pure tone-like sounds when frightened or approaching in a friendly manner. Such considerations might have evolutionary roots, as harsh and rough sounds tend to be associated with the size of the animal producing them. In fact, body mass correlates with vibrating membrane and a more massive vibrating membrane can more likely produce secondary vibrations and hence rough sounds.^3^ Hence, roughness might biologically convey information about the size of the signaller, thus warning about the potential danger and triggering defensive behaviours (though see Gingras et al., 2013).^4^ Recent studies confirmed that aggressive nonverbal vocalizations (roars) cue physical strength in both Western and non-Western cultures (e.g., Kleisner et al., 2021; Raine et al., 2019), thus suggesting that specific nonlinear phenomena, namely roughness, are among those that might predict strength, a physical trait that is associated to potential danger for the listener in case of aggression.

Studies on humans and animals provide additional evidence on the association between roughness and danger/distress situations. For example, Koutseff et al. (2018) have shown that the acoustic structure of baby cries significantly varies between non-painful and painful conditions. In particular, they demonstrated that cries that were associated with pain contained higher levels of roughness than nonpainful cries, suggesting that auditory roughness might provide a biological cue to communicate distress. Relatedly, a magnetoencephalography (MEG) study by Young et al. (2016) demonstrated that the different acoustic profiles of adult and infant cries induce different neural responses that allow the listener to rapidly identify infant cries and thus promote prompt adaptive behaviours. In a large online study, Anikin et al. (2021) investigated the effects on nearly 700 listeners’ perceptions of three psychoacoustic (pitch, timbre, roughness) and three ecological (body size, formidability, aggression) characteristics. The results confirmed that nonlinear vocal phenomena lowered perceived voice pitch, increased voice darkness and roughness, and caused vocalizers to sound larger, more formidable, and more aggressive. Moreover, in the absence of emotional or social contextual information, auditory roughness *per se* has been shown to trigger defensive reactions in humans with respect to nonrough stimuli (harmonic tone; Taffou et al., 2021). For these reasons, infant-directed vocalizations across cultures tend to be less rough than adult-directed ones (Hilton et al., 2021).

The association between roughness and danger is also demonstrated in the context of entertainment, such as cinema. Analyzing horror movie soundtracks, Trevor et al. (2020) found that their acoustic profiles exhibit similarity with screams (i.e., a high level of auditory roughness) and that they are typically perceived as having a negative valence (see also Blumstein et al., 2010). For example, for the famous scene of Hitchcock’s 1963 film, *The Birds*, a horrifying noise was obtained using an early electronic instrument, the Trautonium, which displays a rich set of nonlinear characteristics (Wierzbicki, 2008). Especially relevant for our multisensory perspective are some of the early comments to that movie. For example, noting the affective contrast between speech and noise, one reviewer for the *Christian Science Monitor* observed that the “interplay of brittle humans and predatory birds is developed as a kind of miasmic anti-music, aimed at eye and ear” (Chapin, 1963, p. 16), while the film critic for the *Los Angeles Times* wrote that the bird noise “scratches you like a fingernail across glass” (Scheuer, 1963, p. D13).

Roughness also appears to be an aversive feature of animal sounds (e.g., in seals; Götz & Janik, 2010). The acoustic profile of rumbles produced by African elephants in distressed contexts exhibits increased roughness relative to those rumbles emitted in neutral and positive contexts (Soltis et al., 2011), and roughness-like structure is also present in the vocalizations that are emitted by bats in those contexts that they find distressing (Hechavarría et al., 2020). Many rodents—including shrews, marmots, meerkats, and prairie dogs—emit rough warning calls when danger is imminent (see also Marx et al., 2021, for results on nonlinearities as stress indicators in dog whines). These timbrally “nonlinear” calls are capable of conveying information both about the kind of predator and its behaviour, information of obvious adaptive advantage to the group.^5^

Analyzing the spectrotemporal profile of human screams, Arnal et al. (2015) found that screamed vocalizations contain stronger temporal modulations in the range 30–150 Hz, a range of temporal modulations, generally considered irrelevant for human communication that, as noted above, corresponds to the perceptual attribute of roughness. In the repertoire of humans’ nonphonetic vocalization, screaming is a primary means of increasing signal intensity in order to amplify sensory salience and increase the likelihood of efficient reactions on bystanders who may often lack other situational information about the caller. Screams might initially have evolved for defensive purposes (i.e., to signal potential attack by predators and, at the same time, to discourage predators, e.g., by hopefully soliciting aid from conspecifics; Rohwer et al., 1976).

Interestingly, the evidence shows that those noises produced by friction between various types of surfaces (metal, stones, wood) that have been considered as an “auditory irritant” (Boyd, 1959; see also Ely, 1975) present some of the acoustic features of roughness.^6^ Halpern et al. (1986) confirmed that the unpleasant quality associated with the sound of a solid object scraped across a chalkboard is signalled by acoustic energy in the low-middle range of frequencies audible to humans, while more recent evidence has shown that temporal modulations in the range from 1 to 16 Hz were important predictors for the unpleasantness of sounds (Kumar et al., 2008). Attempting to explain why such sounds are so grating to the human ear, Halpern and colleagues stressed the similarity between the spectrogram of such sounds and of warning cries emitted by macaque monkeys, thus suggesting that the sound might mimic some naturally-occurring, innately aversive event, such as the presence of a predator.

To summarize, the literature reviewed here supports a link between auditory roughness and aversion. In the natural world, rough auditory stimuli signal potential danger or harm, which are likely to be perceived as unpleasant. Intriguingly, the fact that dissonant sounds elicit temporal modulations in the spectrotemporal range that is also exploited to communicate danger suggests that aversion to dissonance might have biological origins. Interestingly, investigating the neural dynamics elicited by auditory roughness, Arnal and colleagues (2019) reported that rough sounds tend to synchronize activity throughout superior temporal regions, subcortical and cortical limbic areas, and the frontal cortex. These neural networks are classically thought to be involved in the processing of aversive stimuli (such as images of attack scenes or mutilated bodies). Together with evidence on roughness-associated synchronized phase-locked oscillatory activity in primary auditory cortex (e.g., Fishman et al., 2000; Fishman et al., 2001), these studies might lead one to hypothesize that the roughness of dissonant stimuli triggers neural synchronization and, in turn, aversive behavioural reactions.

## Surface roughness: Touch and vision

Among the various attributes that characterize surface textures, such as stickiness or friction, roughness is probably the most intensively-studied one, for at least two related reasons. First, roughness strongly contributes to determining the physical or perceptual qualities of surfaces (e.g., Hollins et al., 1993; Hollins et al., 2000), and is typically the dominant axis in perceptual ratings of tactile textures (Hollins et al., 2000; see also Lieber & Bensmaia, 2019, for neurophysiological evidence from primates). Second, it is relatively easy to empirically investigate surface roughness perception using a wide range of perceptual/discriminatory protocols and stimuli (e.g., see Gallace & Spence, 2014; Klatzky & Lederman, 2010, for reviews).

While the perception of auditory roughness is typically conceived of as a temporal process, the tactile perception of surface texture is an essentially spatial or, at least, spatiotemporal property. During the haptic exploration of sandpapers, the spatial distribution of salient geometrical properties, such as the size, shape, density and arrangement of bumps, grooves, and other surface elements, is registered by the static contact of finger skin with the surface, allowing the perceiver to distinguish relative roughness of the size 141 and 192 μm (Hollins & Risner, 2000). The discrimination threshold can be greatly improved (9 and 15 μm, respectively) simply by moving the fingertips dynamically across a surface.^7^ The fingertips have a high density of specialized mechanoreceptors ideal for such a task (Johansson & Vallbo, 1979), as well as a large area within the somatosensory cortex dedicated to the processing stimulation from the fingers relative to other body parts (Gallace & Spence, 2014; Sutherling et al., 1992). As far as movement is concerned, Heller (1989) failed to observe any difference in performance between active touch (i.e., movement of the hand over a surface) and passive touch (i.e., movement of the surface over a stationary fingertip). Furthermore, roughness perception (Bolanowski et al., 1999; Lederman & Abbott, 1981) and roughness discrimination (Lamb, 1983) do not appear to vary as a function of whether active or passive dynamic touch is used. From these findings, it might be concluded that relative motion, rather than hand movements, is what is crucial to the spatiotemporal encoding of texture information.

The distinction between the macro- and micro-geometric elements of surface texture is very relevant to the haptic discrimination of roughness. At the macro scale, early studies using various kinds of surfaces (e.g., sandpapers and plates with randomly arranged conical elements) demonstrated that surface roughness increases monotonically as a function of the spacing between elements that form the texture (Lederman & Taylor, 1972; Taylor & Lederman, 1975)^8^ and that macrotexture perception is a spatial, rather than a temporal, phenomenon (see Lederman, 1974, 1983). More recently, others have shown evidence for small contributions of temporal frequency to the perceived magnitude of macrotextures (Cascio & Sathian, 2001; Gamzu & Ahissar, 2001; Smith et al., 2002), but the evidence predominantly supports a spatial mechanism. Microgeometric elements have spacing between the elements that is below 0.2 mm. Almost a century ago, Katz (1925) suggested that very fine textures were perceived by vibration while more recent work by Bensmaïa and Hollins (2003, 2005) supports a duplex model of roughness perception. According to the latter view there is a transition from the spatial coding of macrotexture to vibratory, and thus temporal, coding at the microscale. Further evidence for a distinct processing of micro and microgeometry also comes from neurophysiological studies (e.g., Roland et al., 1998; Weber et al., 2013).

A recent study by Roberts and colleagues (2020) went deeper into how contact forces affect roughness judgments. Using pairs of periodic spatial gratings in either the fine (320–580 micron) or coarse (1,520–1,920 micron) ranges, they aimed to examine whether, on a trial-by-trial basis, discrimination performance (correct vs. incorrect response) was related to the contact force parameters on that trial. The results revealed that roughness discrimination when sliding was better than when pressing onto surfaces in the fine but, importantly, not in the coarse range (in line with Bensmaïa & Hollins, 2003). Moreover, correct discrimination in roughness judgments was linked to greater normal force in pressing in the coarse but not the fine range. These results therefore confirm that roughness perception depends on the particular properties of the surface texture, with fine spatial textures (spatial dimensions up to a few hundred microns) being discriminated in terms of vibration during sliding, whereas coarse textures (spatial dimensions of several hundreds or thousands of microns) being discriminated in terms of spatial pattern during pressing.

Although people typically assess the roughness of a surface simply by rubbing their finger(s) over it, they can also obtain relevant information by inspecting it visually. Vision and touch are thus assumed to provide information about one and the same external physical property of the perceived stimuli. Although visual and haptic information might simultaneously affect the perceiver, the surface properties of an object are often primarily perceived visually (Schifferstein, 2006; Schifferstein & Cleiren, 2005), and this, in turn, may then guide the tactile system to explore the surface. Thus, although possible, unisensory touch is the more unusual condition, and it often occurs in controlled scenarios (i.e., experimental protocols). Hence, scholars have proposed that the visual system preattentively extracts pattern features that might help to predict the haptic qualities of surfaces, such as grain size, density, or regularity (Julesz, 1984; Julesz & Bergen, 1983). Interestingly, as shown by the study of Van Egmond et al. (2009), visual roughness is not consistently rated as either aesthetically pleasant or unpleasant. Noteworthy here, the ability to detect change in texture visually constitutes an important aspect of object segregation, which plays an evident biological role, for example, in the ability of animals to determine the location of prey in visually complex environments (often complicated by the latter’s camouflage).

The multisensory (especially visuotactile) nature of roughness has been discussed theoretically since early reflections concerning the senses. For example, in his famous treatise on optics, written about a millennium ago, the Muslim mathematician and physicist Ḥasan Ibn al-Haytham, also known as Alhazen, considered roughness (together with smoothness) as one of the 21 “particular properties that can be perceived by the sense of sight,” along with other traditionally visual properties, such as transparency, opacity, shadow, darkness, and size (Sabra, 1989, p. 115a). As observed by Mantovani (2020, p. 133), the terms of Aristotle’s common sensibles have not disappeared from Alhazen’s list, which also included among the visibles “roughness and smoothness,” mentioned by Aristotle in the passage quoted earlier from *De sensu*.

Over the last century, a number of psychophysical studies have proven that the assessment of surface texture typically involves the integration of information from more than one sense (e.g., see Klatzky & Lederman, 2010; Lederman & Klatzky, 2004, for reviews; see Spence, 2020b, on the multisensory texture-related, or rather material-related, Japanese concept of “shitsukan”). A question might arise here regarding the sense that eventually outperforms the others in the assessment of surface texture. The research confirms that both vision and touch are well suited to the assessment of surface roughness, and that both modalities can be used to perform equally well, at least for those tasks involving standard abrasive papers of moderate roughness (e.g., Binns, 1937; Björkman, 1967; Heller, 1985; Lederman & Abbott, 1981). For example, Binns (1937) found no difference between the two modalities in the ordering of a small number of fabrics by softness and fineness. Lederman and Abbott (1981) found that surface roughness was judged equivalently whether people perceived the surfaces by vision alone, haptics, or using both modalities (see also Drewing et al., 2004b; and Bergmann-Tiest & Kappers, 2006, with only subtle differences being reported).^9^ Interestingly, the results from a matching task reported by Lederman and Abbott (1981, Experiment 1), revealed that bimodal matching led to a mean response that was halfway between the responses to the unimodal components, thus suggesting a process of averaging the inputs from the two sensory channels.

Much less clear is the reciprocal influence of touch and vision on the perception of roughness, although the transfer of texture information between touch to vision is evident from birth (e.g., Molina & Jouen, 2001; Sann & Streri, 2007). Lederman et al. (1986) have shown that the nature of the (experimental) task affects the extent to which the input from one sense dominates over the other, with some tasks appearing to be best accomplished by vision (e.g., shape discrimination), and others by touch (e.g., roughness discrimination; see also Heller, 1989; Klatzky et al., 1987; Lederman et al., 1996; see also Phillips et al., 2009, for the effect of object complexity during a unisensory/crossmodal discrimination task). In a study by Ballesteros and colleagues (2005), participants performed different tasks (i.e., a free classification task, a spatial arrangement task, and a hedonic rating task) that required the participants to explore 20 ecological textured surfaces haptically or by touch and vision simultaneously. Besides confirming the key role of roughness as a prominent organizing factor supporting texture perception, the study revealed that the results obtained from the bimodal (haptics and vision) and tactile only exploration were highly correlated. A few years later, in a series of experiments with children (ages 5 and 8 years old), Picard (2007) investigated both intramodal and crossmodal performance in the perception of surface texture using fabric samples. With a relatively easy discrimination task, performance was equivalent across vision and touch. However, when the test stimuli shared similar tactile properties, visual recognition was better than tactile recognition performance. Additionally, Henson et al. (2006) showed that the same texture sample can be evaluated as having different connotative values when experienced in different modalities (somatosensory, visual, or both somatosensory and visual).Taken together, therefore, these findings provide inconsistent evidence concerning generalized biases toward the visual/haptic modality in surface exploration, rather indicating that the sensory information about texture that is considered as dominant depends on the task and on object complexity (see also Whitaker et al., 2008).

Recently, Kuroki et al. (2021) investigated the roles of lower- and higher-order surface statistics (see Portilla & Simoncelli, 2000) in the perception of tactile texture. They created 3D printed, haptic versions of different natural elements, such as stones and leaves, by translating the intensity values of photos of those natural objects as a height map. The contrast difference between complete black (0) and complete white (255) in an image was transcribed to a height difference of 2 mm, with a mean depth of 1 mm. The results revealed that participants failed to discriminate some texture pairs, despite their being well above the haptic discrimination threshold, suggesting that touch differs from vision not only in spatiotemporal resolution but also in sensitivity to high-level surface statistics.

Given that the sense modality (i.e., vision or touch) that is more informative in the perception of roughness varies depending on several factors, it might not be so surprising that multisensory integration research has failed to provide clear evidence of any improvements on discriminative/perceptual performance thanks to the combination of visual and tactile information (e.g., Jones & O’Neil, 1985; Lederman & Abbott, 1981), nor for combinations of tactile and auditory information either (Lederman, 1979). Comparing roughness judgments across unisensory, visual, and tactile, and bimodal conditions, Jones and O’Neil (1985) found no difference between these conditions on performance accuracy, reporting that decision speed was quicker in vision than in touch but that decision speed in the bimodal condition represented the average of these two conditions (see also Cavdan et al., 2021, on softness). In contrast, Heller (1982) observed that multisensory information improved accuracy on a three-alternative smoothness rating task, relative to the unisensory conditions (although, on closer examination, the benefit for the bimodal condition was the result of the observer viewing their hand movements during the task rather than any benefit on the multisensory perception of texture *per se*). Finally, relevant here, a recent study by Ono et al. (2022) has revealed that roughness gives rise to visuotactile interaction in binocular rivalry. In a series of three experiments, participants were exposed to tactile and visual stimuli that differed in roughness (e.g., patches of bathmat or turf and images of the same materials). In the tactile modality, they were presented with one stimulus on each trial (either bathmat or turf), while the two visual stimuli were presented separately visually (i.e., turf and bathmat to right and left eye, respectively). The results showed that the congruent and incongruent tactile stimulation significantly affected the dominant time of visual percepts.

Using a speeded roughness discrimination task, Guest and Spence (2003a) demonstrated that touch was more effective than vision when discriminating smooth surfaces, although this pattern of performance reversed for rougher pairwise discriminations. These results therefore suggest that for roughness assessment, touch can, in fact, be inferior to vision. Meanwhile, the results of another study reported by Guest and Spence (2003b) confirmed that the bimodal integration of information concerning textural roughness occurs in such a way that multiple sensory inputs act as weighted—but potentially redundant—sources of sensory information. Finally, in line with behavioural differences between vision and touch in the perception of surface texture, neurophysiological evidence suggests different, and at least partially independent, neural substrates in the brain for the two systems. For example, the cortical regions underlying tactile texture perception are the primary and secondary somatosensory areas, the posterior parietal cortex as well as other more anterior brain regions such as the prefrontal cortex. The visual processing of texture, on the other hand, involves cortical brain areas that are generally distinct from those that are involved in tactile perception such as the primary visual cortex, the collateral sulcus, and other higher visual areas such as the fusiform gyrus (see Whitaker et al., 2008, for a review). However, the limited neuroimaging research that has been published on this topic to date also suggests that the brain areas involved in determining surface texture are, at least to some extent, shared between the senses (e.g., Eck et al., 2013). Relatedly, Sun et al. (2016) found that brain activations are elicited in the secondary somatosensory area associated with tactile stimulation when looking at glossy and rough surfaces.

Given that in many other multisensory tasks, multisensory integration enhances performance—for example, in the perception of audiovisual speech or other signals (Calvert et al., 2000; Mulligan & Shaw, 1980; Risberg & Lubker, 1978) and of odour paired with taste (Dalton et al., 2000)—it might be surprising that visual and tactile cues act as potentially redundant sources of sensory information regarding the perception of roughness. Thus, one might eventually be tempted to draw conclusions concerning the nature of visuo-haptic roughness perception. In fact, since the visual and tactile modalities seemingly provide independent information concerning roughness across a wide range of perceptual/discriminatory tasks,^10^ and there is no evidence that such information is integrated in order to improve performance, it might be likely that visuo-haptic roughness is an Aristotelian “common sensible”—that is, a stimulus feature that can be perceived independently by (at least) touch and vision. If this were to be the case, then, as suggested by Guest and Spence (2003b), multisensory integration would appear to be an energy-consuming and inefficient means of processing sensory information, as focusing on a single sense modality is typically sufficient to obtain all available data.

To summarize, both ancient theoretical reflections and recent empirical research suggest that vision and touch pick out the same perceptual property when we attribute roughness to the stimuli that we perceive. In a context in which vision and touch are both used to explore the nature of textured surfaces, vision appears to be biased toward encoding pattern or shape descriptions, and touch toward roughness discrimination. The relative weights assigned to the senses appear to be controlled, to a large extent, by attentional processes. Interestingly, a certain tactile dimension of roughness was also present in Helmholtz’s (1954) purely auditory account, when he defined the sound of simple tones, such as those produced by tuning forks, which notably lack roughness, through the tactile quality of being “very soft” (p. 118). However, it is unlikely that roughness in the tactile/visual domain is the same property that is picked-out by hearing as, in the latter context, the spatial dimension, which is crucial for visuotactile roughness, is overridden by temporal dimension, in which the non-linear spectral modulations that elicit auditory roughness occur. By contrast, although tactile roughness can also be determined temporally (as a function of the frequency of the texture-induced vibrations elicited on the skin), empirical findings suggest that the perception of roughness relies on amplitude/intensity rather than frequency/temporal information (Bensmaïa & Hollins, 2003; Hollins et al., 2000; Miyaoka et al., 1999; though see Lieber et al., 2017, for evidence on the temporal processing of finer textures). Additionally, it is likely that roughness depends on the mechanical interaction with the fingertip, as well as the properties of the mechanoreceptors that ultimately transduce the information (e.g., see Manfredi et al., 2014; Scheibert et al., 2009). Hence, it would appear that roughness refers to (at least) two different stimulus properties, one that is primarily perceived in the temporal domain, and the other that is essentially spatial (or spatiotemporal) in nature. In what follows, we move on to investigate whether and how roughness, either temporal or spatiotemporal, can also be referred to in the chemical senses (i.e., gustation and olfaction).

## Food texture, astringency, and (oral-somatosensory) roughness

Although the term *texture* has more commonly been used to describe the surface and appearance of nonfood materials (such as textiles), it is now becoming increasingly popular for consumers and researchers to use the term to describe the perceptual qualities of foods. Features that are perceived by touch are also frequently used to assess the surface texture of food, which is among the factors that contribute most to human’s appreciation of foods, together with flavour and aroma (Bourne, 1982; Spence, 2015). Bourne (1982) defines food texture as “the response of the tactile senses to physical stimuli that result from contact between some part of the body and the food” (p. 259). Several mechanical and structural components contribute to determining food texture, such as hardness, viscosity, elasticity, porosity, juiciness, creaminess, cohesiveness and, relevant for the present article, roughness/smoothness (Chen, 2007). For example, roughness has been included among the textural characteristic of rice (Yau & Huang, 1996) and biscuits (Martínez et al., 2002), with smoothness being relevant for chocolate products (Januszewska & Viaene, 2001).

Mechanoreceptors present in the oral surfaces are capable of sensing and detecting any physical/mechanical stimulus exerted on the tissue surface and are directly responsible for the sensation of food texture. As suggested by anatomical evidence, the oral perception of food texture might be understood in terms of tactile sensation on the fingertips (Foegeding et al., 2015; Gallace & Spence, 2014). In fact, the surfaces of the oral cavity are innervated by exactly the same nerve fibres as the skin of the fingertips, with the possible exception of specific nerve fibres and related Pacinian corpuscle mechanoreceptors (see Bukowska et al., 2010; though see Dong et al., 1993; Haggard & De Boer, 2014). The signal patterns registered by nerve fibres are integrated during higher processing in the brain resulting in the perception of specific basic textural attributes such as smoothness, roughness, and viscosity. At least partially in contrast with the way in which frequency is processed by the cochlea (i.e., tonotopy), specific texture is not coded by a single type of mechanoreceptor, rather by the combination of signals resulting from multiple types of stresses and strains on a given receptive field or population of receptive fields (Foegeding et al., 2015).^11^

In addition, and related to texture perception, the oral-somatosensory attributes of food also give rise to what food science researchers often refer to as “mouthfeel” (e.g., Gawel et al., 2000; Szczesniak, 1979). This term is used to describe the feeling that tasters have in their mouths during, and after, eating a certain food or drink. Foods containing menthol, for example, typically give rise to a cool mouthfeel. Jowitt (1974) described mouthfeel as “those textural attributes of a food or beverage responsible for producing characteristic tactile sensations on the surfaces of the oral cavity” (p. 356).

Among the different mouthfeel characteristics, astringency is generally defined as a “drying,” “puckering,” or, especially relevant here “roughing” mouth sensation (Green, 1993; Lee & Lawless, 1991; Corrigan Thomas & Lawless, 1995). Typically associated with red wine, green tea, and chocolate (Bajec & Pickering, 2008; Jöbstl et al., 2004; Peynaud & Blouin, 1996), astringency results from the exposure of oral surfaces to specific substances, especially polyphenol compounds (Lesschaeve & Noble, 2005). Importantly, astringent substances are thought to reduce lubricity, resulting in a subsequent increase in friction between those oral surfaces that stimulate mechanoreceptors thus giving rise to the “roughing” sensations that are experienced (Bajec & Pickering, 2008; De Wijk & Prinz, 2005; Gibbins & Carpenter, 2013; Lei et al., 2022), and paving the way for the idea that astringency may be a tactile stimulus that is perceived in a manner closely resembling the perception of tactile roughness (Breslin et al., 1993; Green, 1993; Kallithraka et al., 1998). Notably, Breslin et al. (1993) demonstrated that the sensation of astringency can be elicited in the oral cavity in regions where there are no taste receptors, such as the upper lips, lending support to the tactile nature of astringency perception. Finally, as far as tactile roughness is concerned, the perception of astringency reflects a dynamic spatiotemporal process, one that results from the interaction between moving dermal surfaces, such as elicited by tongue–palate and tongue–food interactions (Chen & Stokes, 2012).

Several studies have investigated the way in which astringency is described verbally by wine tasters (and wine writers), providing behavioural support for the relationship between astringency and roughness. When asked to list all of the words that they would use to describe astringency, the participants in one study reported by Vidal et al. (2015) defined it as a rough or dry sensation, felt in the mouth, palate, and tongue when, or after, drinking wine.^12^ When consumers were asked to focus on the sensations they felt when drinking an astringent wine, the terms that were most frequently mentioned were dryness and roughness. In a similar study, Piombino et al. (2020) had their participants describe the astringency of Italian red wines from 11 cultivars in terms of several subqualities, such as dryness, bitterness, harshness, velvetiness. The study confirmed that roughness plays a key role in several definitions of astringency, in which it is linked to dryness and harshness due to the lack of lubrication and feeling of dehydration in the mouth (see also Linne & Simons, 2017).

In addition to behavioural studies, research has investigated the physiological and physical mechanisms affecting the perception of astringency, including saliva composition, flow rate, and the disruption/alteration of the salivary pellicle (Gibbins & Carpenter, 2013; Upadhay et al., 2016). Recently, Lei et al. (2022) demonstrated that when encountering tannic acid solution, the roughness of the salivary pellicle increases significantly, and lubrication performance is impaired due to the significant effect of tannic acid molecules on the structure of the salivary pellicle itself. Hence, astringency is generated by the increase of intraoral friction due to the structural changes in salivary pellicle caused by polyphenolic molecules introduced into the oral cavity.

Finally, it should be noted that roughness can also be related to the graininess, or grittiness, of foods. Graininess is one of the fundamental textural features that can negatively affect the overall perception of foods (Muñoz & Civille, 1987; Tyle, 1993). For example, chocolate with a particle size above 35 μm is usually perceived as gritty or coarse in the mouth, and it is not accepted by consumers, nor is ice creams with ice crystals larger than 40 μm (Breen et al., 2019; De Pelsmaeker et al., 2020; Puleo et al., 2020; Servais et al., 2002). Interestingly, Li and Montell (2021) recently demonstrated that the *Drosophila melanogaster* is endowed with the ability to discriminate smoothness versus grittiness of food texture and uses this information to decide whether a food is appealing or not. One other angle here concerns the way that certain textures may be associated with specific taste qualities, as in the case of cotton candy/candy floss (see Spence et al., 2019).

The literature that has been reviewed here suggests that roughness is a relevant percept in the domain of gustation. Conceived of in terms of tactile perception, roughness is used to describe the texture of food, and it is also often associated with an astringent mouthfeel. Interestingly, the tactile opposite of roughness—namely, smoothness—is considered among the factors contributing to determining the sensation of creaminess, which can also be opposed to astringency (Upadhyay et al., 2020). Physiological studies have provided a biological basis for the association between astringency and roughness, revealing that astringency is driven by the roughness and wettability of the salivary pellicle and showing, at the same time, that increased intraoral friction is an inevitable consequence of astringency. Sharing a number of behavioural and biological features with haptic roughness (including its spatiotemporal nature), it remains unclear, however, whether oral-somatosensory roughness is to be conceived of as a different kind of roughness perception, or simply as a different context in which the same (i.e., tactile) perceptual property is apprehended. To shed light on this question, and on the different possible sensory components of roughness and their presence across sensory domains, we will take a critical look at the crossmodal literature on roughness perception, in an attempt also to answer one of the questions that we started with, namely, whether the senses are integrated into a unitary roughness judgment. However, before doing this, we summarize the most relevant aspects of the literature on unisensory roughness, trying to draw some tentative conclusions concerning the different kinds of perceptual properties that are referred to with the term *roughness* in different sensory domains.

## Interim summary: Roughness—A common word for discrete stimulus properties?

**Fig. 2:**
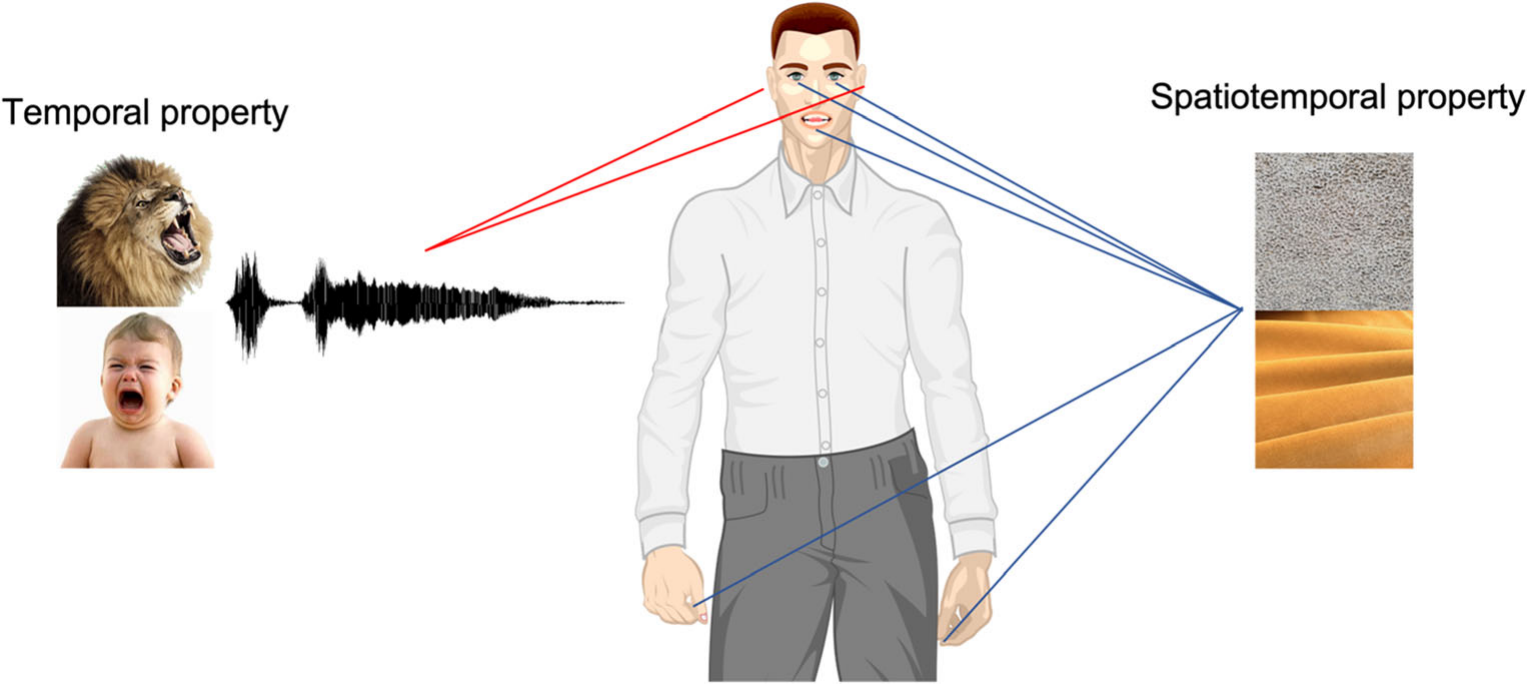
Representation of the main different meanings of the term *roughness*—that is, temporal/spatiotemporal property, and their relationship(s) with different sensory modalities

Based on the literature that has been reviewed here, we might propose to group the different perceptual features that emerged as characterizing roughness in different sensory domains (i.e., auditory, visual, tactile, and oral-somatosensory) into two alternative notions of roughness. On the one side, a temporally perceived stimulus dimension, which essentially characterizes auditory musical (i.e., dissonances) and nonmusical stimuli (i.e., speech, human and animal vocalizations, environmental sounds). In this context, roughness can be thought of as a biologically salient feature that signals a potentially dangerous or harmful situation/stimulus; is typically perceived as unpleasant; triggers attentional behaviours and elicits aversive reactions in the listener. On the other side, we might postulate the existence of a spatiotemporal stimulus dimension, which can be attributed to visual, tactile, and oral-somatosensory stimuli. In this context, roughness tends to be perceived as unpleasant (though to a certain extent appreciated in specific contexts, e.g., wine tasting) and it is elicited by the combination of the static (spatial) exploration of surface texture and the dynamic interaction between dermal surfaces in motion (temporal), such as finger-surface, tongue-palate, and tongue-food (see Fig. 2).

The intimate relationship between vision and touch during the active exploration of surface texture suggests that both senses are picking out the same “tactile” perceptual property. Moreover, anatomical and physiological evidence suggests that a mouthfeel intimately linked to roughness (i.e., astringency) may well be considered a tactile sensation. Hence, we might tentatively answer one of the questions we started this review with, by stating that people use the word *roughness* to refer to (at least) two different phenomena across the senses, a primarily temporal and auditory one, and a primarily spatio(temporal) and (visuo)tactile one (see Table 1).

**Table 1:** Comparison between the temporal and spatiotemporal notion of roughness in terms of sensory domains involved, relevant stimuli, processing mechanisms and their pleasant (P), unpleasant (U), unclear (?) effects on the perceiver

	Sensory domain	Stimuli	Underlying mechanism	Pleasant/ unpleasant
Temporal roughness	Hearing	Sounds (e.g., dissonances) Nonlinear animal and human vocalizations (e.g., distress cries, roars)	Perception of beating at frequencies in the range 15–300 Hz, reaching a maximum at a frequency of around 70 Hz	U
Spatiotemporal roughness	Touch	Surface texture	Tactile perception of microgeometric elements that determine surface texture (e.g., bumps, grooves)	U
	Vision	Surface texture	Visual perception of macrogeometric elements that determine surface texture (e.g., patterns or shapes)	?
	Gustation	Food and beverages (e.g., red wine, green tea, chocolate)	Exposure of the oral surfaces to specific substances, especially polyphenol compounds, which give rise to the typical mouthfeel characteristic of oral astringency	P/?

**Fig. 3:**
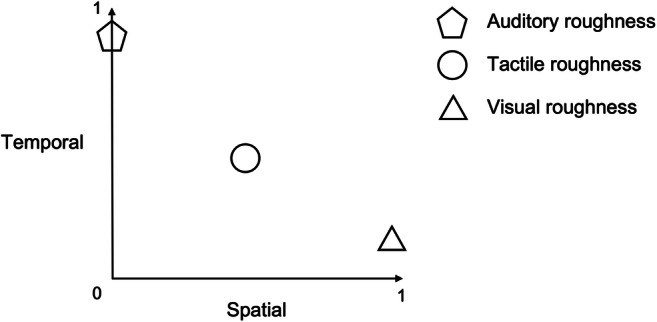
Auditory, tactile, and visual roughness and their relationship with the spatial (*x*-axis) and temporal (*y*-axis) dimensions. The *x-* and *y-*axes range from 0 (i.e., aspatial and atemporal, respectively) to 1, exclusively spatial and temporal, respectively. Visual roughness is mostly but not exclusively spatial due to the role that dynamic exploration (e.g., saccades) might have in perceiving visual roughness

A further distinction might be introduced to differentiate between visual and tactile perception of textural roughness, which can be conceived as (mostly) spatial and spatiotemporal, respectively. Thus, roughness perception ranges from an essentially temporal (and nonspatial) phenomenon (i.e., auditory roughness) to a spatiotemporal one (i.e., the tactile exploration of surface texture). Visual roughness cannot be considered as exclusively spatial given the role that dynamic exploration (e.g., saccades) can sometimes play in helping to determine visual roughness (e.g., Jacobs et al., 2010; see Fig. 3).

One might object that—with the notable exceptions of the Finnish architect Juhani Pallasmaa, who vividly recounts the urge that he once experienced to feel architecture using his tongue (Pallasmaa, 1996, p. 59), and of the British writer and artist Adrian Stokes (1978), who once wrote of the “oral invitation of Veronese marble” (p. 316)—there are very few occasions when we can both see and feel same shape or texture/roughness.^13^ Experiencing materials both visually and haptically, in fact, could constitute a special case limited to experimental contexts in which peculiar materials, such as sandpaper or pilled fabrics, are chosen as stimuli. If such an objection seems to faithfully reflect the way in which we ordinarily experience roughness, it should not override the fact that, although in most cases we cannot simultaneously “touch-and-see,” any additional visual information that one could eventually obtain regarding the roughness of a surface would likely inform on the same textural property inspected haptically (and vice versa). Such a claim might be supported by neuroscientific evidence suggesting crossmodal interactions in early sensory cortices between visual and haptic textural information (Eck et al., 2013, see also Sathian & Lacey, 2022, for a recent review). By contrast, we cannot touch or see the sound we hear as rough to obtain additional data concerning its roughness, because auditory roughness is a temporal property, while vision/touch can inform on spatiotemporal roughness (i.e., surface texture).

In summary, it would seem that while the majority of experimental studies have adopted very particular classes of stimuli when investigating roughness perception, our conclusion can be generalized stressing the fact that, even if we do not typically touch or lick the bricks of a building (*pace* Pallasmaa and Stokes), any tactile information we could get from such sensory experience would be essentially related to what we visually experienced as rough. This, in turn, allows us to make a further clarification about the nature of astringency which, being generated by the mechanical contact between dermal surfaces and food/beverages, is considered to be tactile though it does not provide information on the surface texture of food/beverages.

## Crossmodal interactions in roughness perception

Almost a century ago, the eminent early psychologist Heinz Werner (1934) wrote: “Le lien intime des sens, l’existence de qualités intersensorielles comme la clarté, l’intensité, la rugosité, etc., tout cela est fondé sur le fait que l’organisme psycho-physique réagit dans sa totalité, avant toute séparation en sphères distinctes de sensibilité”^14^ (p. 202). According to such a *synaesthetic* view of perception, roughness is mentioned as one of the allegedly amodal or intersensory qualities that could pave the way for crossmodal perception. In this section, we take a closer look at the literature on the crossmodal perception of roughness. Starting from the audio/visuotactile contexts, we examine several other combinations of sensory domains, also with the aim of investigating the multisensory experience of, or the crossmodal influence on, texture.

### Audiotactile

Given the salience of roughness to the senses of touch and hearing, one might expect audiotactile roughness to be particularly relevant in the literature and in the psychology of perception. Indeed, the Hungarian psychologist Pál Harkai Schiller, also known as Paul von Schiller, noted long ago that those sounds—noise bursts or tones that are repeated at regular intervals—may affect the tactile perception of roughness (von Schiller, 1932). Such a claim might be interpreted in one of two ways. First, as an indication about how auditory and tactile information are integrated multisensorially in the perception of roughness. Second, and more in line with the scope of the present paper, as indicating the possibility that sensory roughness might be perceived (independently) in both the auditory and tactile domains, thus opening up the discussion to issues concerning crossmodal associations. While several studies have been published in an attempt to demonstrate auditory-tactile integration in the perception of roughness, apparently less effort has been put in the investigation of the crossmodal association between auditory and tactile domains mediated by roughness.

In the context of multisensory integration, a number of early studies reported that when we perceive surface texture, tactile cues tend to dominate auditory cues (Heller, 1982; Lederman, 1979). Given that touching rough surfaces rarely produces significant sounds in our daily lives, one would also expect roughness perception to be almost completely determined by tactile cues. This seems at least partially confirmed by the results of a study by Altinsoy (2008), which showed that when auditory, tactile, audio-tactile cues are available, the auditory modality tends to be less informative regarding the roughness of surfaces than the tactile modality. However, several studies have shown that, although allegedly dominated by tactile information, the perception of roughness is also altered by touch-produced sounds. Findings by Jousmäki and Hari (1998) and Guest et al. (2002) both demonstrated that the perceived roughness of palmar skin was altered by the feedback of the sound produced by rubbing the hands together (see also Katircilar & Yildirim, 2022). Yau et al. (2009) investigated whether frequency channels are perceptually linked across the senses of audition and touch. In a series of psychophysical studies, they demonstrated that performance on a tactile-frequency-discrimination task is impaired when an auditory distractor is presented with the tactile stimuli, but only if the frequencies of the auditory and tactile stimuli were similar (See also Bernard et al., 2022, on the auditory and haptic perception of rhythm).

In the context of crossmodal associations, Hamilton-Fletcher et al. (2018) compared sound-touch correspondences in sighted and blind adults, testing whether visual experience would influence the strength and direction of sound-touch crossmodal associations. Although some correspondences were reduced or absent in blind adults (namely, pitch shape), the results show that other correspondences are maintained in the absence of visual experience (pitch size, pitch weight), and others appear to be stronger in the blind than in the sighted (pitch-texture, pitch-softness). In particular, compared with sighted controls, early and late blind persons tended to associate low pitch with rough textures and high pitch with softness, and high pitch with smooth textures. Similar associations were also documented in sighted individuals by Eitan and Timmers (2010) who, however, studied verbal associations between sounds and words like *rough* and *smooth* (see also Etzi et al., 2016, on the association between the nonsense words *bouba* and *kiki* and tactile smoothness and roughness). In the study by Murari and colleagues (2015), participants listened to excerpts from the Western classical repertoire—that is, six major (Experiment 1) and six minor (Experiment 2) 30-s lasting fragments and were asked to express subjective ratings on seven sensory factors, such as soft–hard, cold–warm, rounded–angular, and 13 adjective couples (e.g., active–passive, masculine–feminine, gentle–violent). Whereas the former were sensorially presented to participants using materials such as pieces of wood/foam (hard–soft) or sandpaper (roughness), the latter were only verbally presented. The results demonstrated that participants tend to match higher values of roughness to minor tonality, with some possible exceptions (e.g., Mozart, which obtained similar matching for both minor and major excerpts). Finally, Wallmark and Allen (2020) studied preschoolers’ crossmodal correspondences involving timbre (i.e., smooth vs. rough) and suggested that crossmodal timbre associations may appear early in human development (e.g., prior to substantial linguistic influence via musical training).

Taken together, these studies provide evidence that auditory roughness affects the perception of tactile roughness in the context of multisensory integration and suggest that tactile roughness is crossmodally associated to low pitch with no effect of visual experience.^15^ Behavioural results from a study by Suzuki and colleagues (2008) suggest that auditory and tactile roughness processing might be based on common neural mechanisms, with neuroimaging studies on the integration of touch and audition in early stages of information processing (Murray et al., 2005) providing additional support for the idea of roughness-mediated crossmodal associations. However, it is worth noting here that while multisensory integration may involve sounds produced by the object during tactile exploration (which might affect tactile roughness perception it might not independently be perceived as rough), crossmodal association is typically tested with auditory stimuli that are not generated from the exploration of an object (e.g., musical notes or timbre). Hence, to date, based on the published literature, the issue remains open.

### Visuotactile

With respect to audiotactile associations, the crossmodal correspondences between visual features, such as hue, and tactile properties has been far less investigated to date. Ludwig and Simner (2013) investigated the associations between colour and haptic sensations—namely, roughness and hardness. Participants had to match haptic stimuli to colours. Their results suggested a linear association between the dimensions of softness/hardness and smoothness/roughness with brightness. As haptic stimuli became either softer or smoother, they were matched to brighter colours. The authors also found significant effects of saturation, but only for the youngest group tested. Smoother and softer stimuli were associated with colours of higher saturation compared with rougher and harder stimuli. Yellow, pink, and white were chosen significantly more frequently for the smoothest stimulus, while black and brown were chosen significantly more often for the roughest stimulus.

In a study by Slobodenyuk et al. (2015), the participants matched haptic sensations—namely, roughness, hardness, heaviness, elasticity, and adhesiveness—to colours. Haptic sensations were rendered via a haptic device that allowed for the reproduction of sensations at different intensities (i.e., from 1 to 6, low to high, respectively). The results showed that, regardless of the particular sensation, the least intense haptic stimuli were associated with bright colours while the most intense haptic stimuli were associated with dark colours. Moreover, the participants tended to match rougher, harder, and heavier sensations to red and purple-red hues, while they matched softer sensations to yellow and green-yellow hues. In a subsequent study by the same group (Jraissati et al., 2016), a group of Arabic participants was asked to match haptic terms to 64 Munsell colour patches. Eleven pairs of opposed haptic terms were used, corresponding to the following English paired terms: soft/hard; smooth/rough; sticky/nonsticky; supple/rigid; elastic/stiff; viscous/fluid; light/heavy; warm/cold; thin/thick; dry/humid; pointy/round. Sixty colours were selected as stimuli from the outer surface of the Munsell colour solid, four additional stimuli were achromatic. Regarding roughness, the results confirmed findings by Ludwig and Simner (2013), showing that participants tend to associate black and brown to roughness, while pink and white are associated with smoothness (see also Wright et al., 2017).

In summary, the studies considered here suggest that haptic sensations (or terms) of roughness are associated with dark colours (i.e., black and brown), while sensations (or terms) of smoothness are associated with light colours (e.g., pink, white). Similar patterns were also observed for saturation in three of the studies mentioned, where opposite haptic sensations were matched to opposite saturation levels (Ludwig & Simner, 2013; Slobodenyuk et al., 2015). However, according to the important distinction highlighted by Stevens (1957), roughness and saturation can be considered as prothetic dimensions, while hue is likely a metathetic dimension (see Pridmore, 1992, for hue as a circular dimension). While the former are quantitative perceptual continua, with a clear “more than” and “less than” end, the latter are well-structured and organized perceptual dimensions without necessarily having a “more than” or “less than” end.^16^ Thus, it perhaps makes it (more) difficult to establish a criterion for the correspondence between hue and roughness.

### Audiovisual roughness perception

**Fig. 4:**
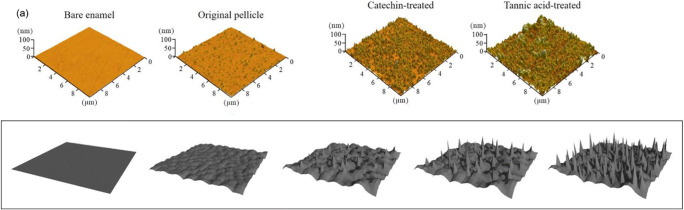
Upper row. Morphology of the bare enamel and the three salivary pellicle modified samples in Lei et al. (2022). Lower row. Visual stimuli used by Giannos et al. (2021) to study crossmodal associations between melodies harmonized with different degrees of roughness and 3D surface textures

Several recent studies have examined the relationship between auditory roughness and shapes, showing that listeners tend to match more dissonant sounds to spikier and rougher objects/shapes (and vice versa; Liew et al., 2018; Liew et al., 2017). Giannos et al. (2021) extended the investigation from isolated sounds to the harmonic context, hypothesizing that nontonal and highly dissonant harmonic stimuli would have been associated with rough images, while more consonant stimuli would be associated with the images of low visual roughness. To test this hypothesis, they harmonized a fixed melody in seven different styles, including highly tonal, non-tonal, and random variations, asking their participants to match the melodies to images of variable roughness (i.e., black and white 2D and 3D images that represented surfaces with different degrees smoothness/roughness). Interestingly, these artificially created images resemble the aspect of the salivary pellicle when modified by astringent substances (see Fig. 4). The results confirmed that auditory dissonance was highly correlated with visual roughness.

In a series of two experiments, Wallmark et al. (2021) asked whether the timbre of a musical note (an acoustic prime) would affect the subsequent visual perception of, in the first experiment, brightness (dark–bright dimension) and, in the second experiment, both brightness and spatial texture (smooth–rough dimension). To this end, they used a speeded-response paradigm in which the participants had to identify a shift in roughness/brightness between two consecutively-presented target squares of subtly contrasting levels (rougher/brighter, smoother/darker, or the same). In Experiment 2, before the presentation of the target square, the participants could be exposed to sounds that varied in terms of their roughness (smooth/rough). For visual stimuli, they used close-up photos of sandpaper patches of slightly contrasting grit sizes: baseline, medium roughness (100-grit 3M wood sandpaper); low roughness (150 grit); and high roughness (50 grit). For sounds, they used a sine wave and a sawtooth wave. They found that, although presenting task-irrelevant tones (crossmodally congruent and incongruent) sped up visual responding relative to a no-sound control, there were no effects of congruency on accuracy or reaction time. Modest evidence was found that timbres increase response bias in a semantically congruent manner when participants identify visual stimuli (e.g., when a “rough” saw-tooth wave accompanies the second of two identical spatial textures, the “rough” sound increases the probability of judging the second texture as rougher), thus suggesting that rough sounds may increase judgments of roughness of the visual percepts (see also Wallmark & Allen, 2020).

There might be several ways to explain why (and how) the relationship between rougher objects and harsher sounds exists. In terms of semantics, it seems quite straightforward to observe that the same term, namely roughness, is used in both the visual and auditory domain to describe certain features of sensory stimuli, and this might likely lead to pairings between the two stimuli. In this regard, Spence and Di Stefano (2022a) extended the notion of “harmony” beyond hearing, thus including also those pairings of crossmodal sensory stimuli that are pleasurable and go well together. However, if the processing of stimuli in the auditory and visual systems were to be unrelated, then crossmodal semantic conventions would lack a simple biological explanation, thus requiring a different explanation. A more direct causal relationship that might explain the link between rough objects and rough sounds relates to the friction between objects with different types and levels of microgeometry that tend to produce harsh noises. In other words, based on statistical learning, people would have experientially acquired to associate harsh sounds with rough objects. Alternatively, one might also put forward an explanation based on affective/emotional mediation (Spence, 2020a). Given that angular/rough shapes have been associated with threat, danger, and negative concepts in general (Bar & Neta, 2007; Palumbo et al., 2015), and, as noted above (see section Auditory Roughness in Human and Animal Vocalization and Communication), auditory roughness evokes potential danger as well, the crossmodal association might also be explained in terms of perceived affective features of the stimuli in each sensory domain. This hypothesis might also account for results on sound symbolism, in which spikier shapes are associated with the vocal roughness of (pseudo-)words (Lacey et al., 2020).

**Fig. 5:**
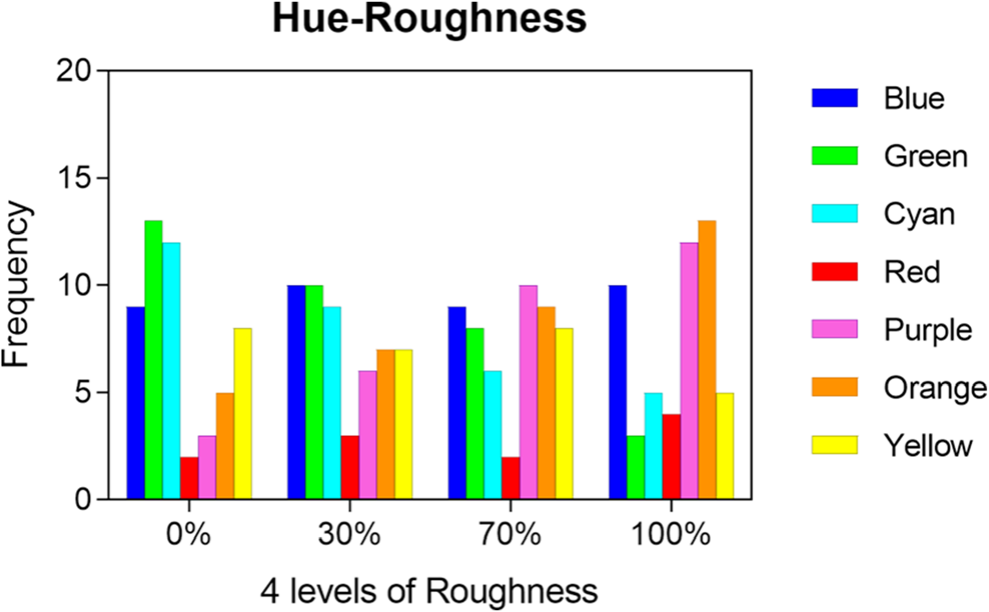
Plot depicting the frequency with which the different hues were selected for different values of roughness. The seven colors are shown along the *x*-axes, each color has four different values corresponding to the four levels of roughness (Reprinted from Sun et al., 2018). (Color figure online)

However, if the mediation of emotion might work well as far as accounting for the association between rough shapes/objects and harsh sounds is concerned, it seems less useful when it comes to interpreting consistent findings on the audiovisual association between auditory roughness and colours. In fact, when the participants were asked to match colours with sounds that varied in terms of their roughness, they tended to match dark colours with very rough sounds, while less rough sounds were matched with light colours (Sun et al., 2018). In particular, roughness has been associated with green, cyan, purple, orange, but not with red and yellow (see Fig. 5). Additionally, roughness has also been associated with low lightness in sound-lightness mappings. These findings apparently could not be explained in terms of emotional mediation, as previous literature suggested that colours that have been associated to rough sounds in the study, e.g., such as green and purple, elicit a pleasant effect on the beholder (e.g., Valdez & Mehrabian, 1994).

However, according to the findings reported by Palmer et al. (2013), red and yellow are generally associated with pleasant emotions (happiness), thus at least partially converging with Sun et al.’s (2018) study, in which the participants did not match those hues to roughness. The association between low lightness and roughness might be in line with Palmer et al.’s study. Therefore, given that the association between emotions and hues occurred in experiments based on different protocols, a number of methodological issues arise (e.g., regarding the use of different colour samples or the terms used to characterize participants’ responses) that make it hard to provide a unique interpretation for different findings. For example, while “pleasantness” universally identifies a positive reaction to a stimulus (e.g., a colour), it still leaves open the question about what elicits such an association. Some people, for instance, may associate pleasantness to a colour that they perceive to be relaxing, whereas others may put forward the same association of that hue as being somehow exciting. Thus, whether used for characterizing color samples or for comparing (ranking) colors, single “emotional” terms, such as *pleasantness*, even in the case of participants quantifying it assigning a value, might not be reliable in clearly assessing emotions in crossmodal association. Moreover, strong differences might exist in the emotional response to felt textures depending on individual factors. However, Wallmark et al. (2021) claim that the use of the term *rough* to talk about musical timbre and visual objects might be more than just be an arbitrary linguistic convention: “Perhaps we see brightness and spatial texture not just through eyes, but also (albeit faintly) through timbrally attuned ears” (p. 16). This drives us back at the core question at stake here, i.e., the possibility for intersensory, or amodal, stimuli quality to exist (see Spence & Di Stefano, 2022b).

Finally, here, it is worth mentioning the research that has focused on the crossmodal influences of unpleasant sounds and visual stimuli. For example, Cox (2008) demonstrated that images that are congruently associated with horrible sounds reliably make people perceive the sounds themselves as more horrible than visually unassociated images or a control. More recently, Samermit et al. (2019) presented aversive sounds either concurrently with their corresponding original video (i.e., the video of the action that produced the sound) or with a positive attributable video, which showed a “positive” action that could have allegedly produced the sound. When aversive sounds were paired with their original videos, participants rated the sounds as producing more discomfort, being more unpleasant, and causing more intense bodily sensations than when they were presented alone. Conversely, when the same sounds were paired with their positive attributable videos, participants rated them as producing less discomfort, being less unpleasant, and causing less intense bodily sensations than when they were presented alone. Although consistently showing the effects of visual information on perceived features of auditory stimuli, a general *caveat* should be made here regarding the implications of such studies for roughness perception. Even if the auditory stimuli used in these studies are consistently rated negatively (e.g., unpleasant, horrible, grating), and rough auditory stimuli are judged negatively as well, the factor “roughness” was not directly controlled in the cited experiments.

### Auditory and taste/gustation

**Fig. 6:**
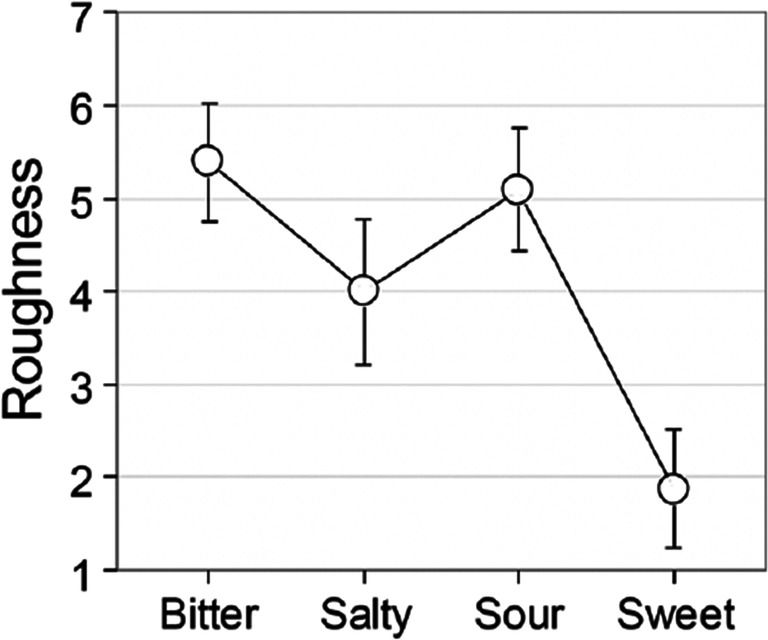
Participants’ selections for auditory roughness in response to basic taste words from a study by Knöferle et al. (2015)

Several studies demonstrated that people systematically map a series of psychoacoustic and musical properties onto basic tastes (see Knoferle & Spence, 2012, for a review).. Findings by Knöferle et al. (2015) showed that the bitter taste was mapped to rough, slow, and low-pitched sounds, whereas sweet tastes were mapped to high-pitched, smooth, and continuous sounds. These associations may be explained in terms of the hedonic account, according to which individuals match tastes that are perceived to be unpleasant (e.g., bitter) with sounds that are judged to be less pleasant, and tastes that are considered more pleasant (e.g., sweet) with sounds that are liked more. The relative mapping of sweet taste onto low values of auditory roughness (see Fig. 6), for example, can be explained by the fact that sweet taste (of moderate intensity) is a pleasant and rewarding stimulus (Moskowitz et al., 1974) and auditory roughness is negatively correlated with sensory pleasantness (Zwicker & Fastl, 2006). However, it should be noted that taste mappings in another auditory dimension that is strongly correlated with pleasantness, namely auditory sharpness, did not follow a hedonic matching account.

Wang et al. (2021) conducted an online study involving a very large sample of participants (*n* = 1,819) to determine the acoustical/musical attributes that best match saltiness. Based on the previous literature on crossmodal correspondences involving saltiness, thirteen attributes were selected to cover a variety of temporal, tactile, and emotional associations. The results of this study revealed that saltiness was associated most strongly with high auditory roughness.^17^ In terms of emotional associations, saltiness was matched with negative valence, high arousal, and minor mode. Given the transitivity of certain crossmodal correspondences (e.g., Deroy et al., 2013), the association between saltiness and roughness, on the one hand, and saltiness and high arousal, on the other, might allow one to conclude that roughness and high arousal are associated as well. In fact, according to literature reviewed in the section Auditory Roughness in Human and Animal Vocalization and Communication, auditory roughness tends to trigger attentional responses and elicits aversive reactions from listeners. Thus, rather than hedonic matching, here it would seem that the emotional association may be the underlying mechanism prompting the association of auditory attributes to saltiness, such as negative valence, minor mode, high arousal, and a high degree of auditory roughness.

### Olfaction and touch

Demattè et al. (2006) carried out two experiments designed to investigate the nature of any crossmodal interactions between olfactory cues and tactile perception, by exploring the possible effect of the presence of different odours on the perception of fabric softness. The participants were presented with fabric swatches and had to rate the perceived softness using discrete values from 1 (*soft*) to 20 (*rough*), while an odor (pleasant or unpleasant) or clean air was delivered directly to their nostrils. The results revealed that pleasant smell modulates roughness—that is, fabric swatches were judged as feeling softer in the presence of a pleasant odor (lemon in Experiment 1, lavender in Experiment 2) than in the presence of an unpleasant animal-like odour. That said, Nishino et al. (2011), have reported inconsistent findings on the influence of odors, i.e., rose, sandalwood, on haptic perception (i.e., of stiffness and roughness).

Koijck et al. (2015) hypothesized that the perception of tactile roughness may be affected by the presence of ambient odour. To test this hypothesis, they conducted two experiments. In a first study, they investigated the influence of ambient chemosensory stimuli with different roughness connotations on tactile roughness perception. In addition to a pleasant odor with a connotation of softness, they also included a trigeminal stimulant with a rough, sharp or prickly connotation (ethanol). Contrary to their expectations, however, they found no significant interaction between chemosensory stimulation and perceived tactile surface roughness. They argued that ambient odors may be less effective in stimulating crossmodal associations, since they are not perceived in synchrony nor necessarily in close spatial proximity to the tactile stimuli. They thus carried out the second experiment, modifying the setting including both pleasant (lemon) and unpleasant (indole) odorants that are known to affect tactile perception under certain conditions. In this second experiment, the researchers found no significant main effect of chemosensory condition on perceived tactile roughness. As the authors observed:

The absence of an effect for pleasant odors is not surprising, given the fact that previous studies showed that pleasant odours on their own only showed a weak tendency to induce a tactile bias (Croy et al., 2014; Demattè et al., 2006) and, at best, show a significant effect when contrasted with unpleasant odors (Demattè et al., 2006). The absence of an effect for unpleasant odors is somewhat unexpected, given the fact that unpleasant odors have previously been found to bias tactile perception of roughness (Demattè et al., 2006) and unpleasantness (Croy et al., 2014). (Koijck et al., 2015, p. 17)

The null result might be due to a wide range of factors, such as the fact that none of the chosen odorants is either inherent or normally experienced congruently with sandpaper, which likely lacks typical inherent smell. By contrast, the stimuli used in Demattè et al.’ (2006) study (fabric swatches) might be more likely to be associated with pleasant smells they typically release when they are bought or freshly washed.

### Touch and gustation

Almost a century ago, the Italian Futurist Filippo Tommaso Marinetti was one of the first to think creatively about the importance of deliberately combining touch, and tactile stimulation, while eating (Marinetti & Fillia, 1932/Marinetti, 2014; see Valentini, 1998). According to Marinetti, the perfect meal demands correspondence between the setting of the table and the dish. Flavors and sensations, especially perfumes, are experienced simultaneously and harmoniously. According to Marinetti, cutlery was to be banned at the table to allow tactile, pre-gustative enjoyment. For example, one dish *Aerovivande* consists of three ingredients (an olive, a candied fruit, and a piece of fennel) and a rectangular surface on which three fabrics with different textures are attached (Valentini, 1998), all of this complemented by floral scents. Interestingly, such trends have subsequently been followed up recently by chefs and experience designers in a number of countries (Spence, 2017).^18^

Interestingly, contemporary research but also contemporary gastronomic practices, are increasingly finding value in a number of the Futurists’ ideas, crazy though they may have seemed at the time (see Spence, 2017). Studies have investigated the mutual influence between touch and taste (gustation) using different foods and surface textures. In the study by Piqueras-Fiszman and Spence (2012), participants tasted digestive biscuits and separately yoghurt, from yoghurt containers that either had a smooth or a sandpaper rough outer surface texture. Pieces of biscuit tasted from the containers with a rougher surface feel were rated as crunchier and harder than those tasted from the normal smooth-sided yogurt containers instead. Notably, no such influence was observed when tasting the yoghurt, leading the researchers to suggest that there must be limiting conditions on the foodstuffs (or food attributes) that may be influenced by the attributes of touch. Another study conducted by Biggs et al. (2016) involved biscuits. In this case, caramelized biscuits were served on two plates of the same shape, one having a rough and grainy surface texture, the other a smooth and shiny texture instead. Biscuits taken from the rougher plate were rated as crunchier and rougher than those sampled from the smooth plate. In a subsequent study, both jelly babies and biscuits were served from textured plates. In this case, the jelly babies were rated as feeling significantly chewier (as opposed to crunchier) when served in the rough plate. In other words, it seemed as if the rougher feel accentuated whichever textural property happened to be dominant in the food experience itself. Finally, in a cross-cultural study involving participants from the East (China, India, and Malaysia) and the West (USA), Wan et al. (2014) found that both the smooth and rough texture patches (presented visually) were strongly associated with the salty taste. However, in that study, authors used images of surface textures as rough/smooth stimuli and terms for taste stimuli (bitter, salty, sour, sweet, and unmami), and thus crossmodal associations might have been influenced or mediated by visual/cognitive factors.

Similar studies have been conducted to assess the effect of surface textures on beverage perception. Wang and Spence (2018) had their participants (*N* = 60) evaluate a red dessert wine whilst simultaneously manually touching either a swatch of velvet or sandpaper. The participants first smelled the wine while touching each material. They rated the aroma of the wine in terms of its orthonasal features (including its intensity, acidity, and fruitiness) and pleasantness. Next, the participants tasted the wine while stroking each material. The wine was rated in terms of its acidity, sweetness, tannin level, and pleasantness. The results demonstrated a significant effect of tactile roughness on aroma/taste judgements. In particular, the aroma of the wine was judged to be significantly fruitier when the participants simultaneously touched the velvet rather than when they touched the sandpaper. When it came to tasting, the wine was rated as significantly sweeter and more pleasant when the participants touched the velvet rather than the sandpaper. These results imply that product-extrinsic surface textures can influence not only mouthfeels, but also orthonasal olfactory evaluations as well.

A couple of other studies used similar paradigms to test the effect of surface texture on the perceived properties of beverages. Using mineral water, Risso et al. (2019) demonstrated that the beverage was perceived as fresher, more pleasant, and lighter when contained in plastic cups than when it was contained in cups that had been covered with rougher surfaces (i.e., sandpaper or satin). Using coffee, Carvalho et al. (2020) demonstrated that the surface texture of the coffee cup affected people’s perception of the flavour of specialty coffee, with a strong main effect on the mouthfeel aspects of the evaluation of aftertaste. In particular, the coffee was perceived as tasting sweeter when sampled from a cup with a smooth, as opposed to a rough, surface. It was also rated as more acidic from the rough cup than when tasted from the smooth cup. Given that in such cases what participants feel is not linked to what they taste, most of the above findings might be explained in terms of metaphorical crossmodal priming, assuming that touching smoother surfaces (e.g., velvet) is typically perceived as more pleasant that touching rougher ones (e.g., sandpaper) as well as more fruity and sweet aromas/tastes are generally perceived as more pleasant.

## Was Aristotle right, after all? On the multiple meanings of roughness across the senses

Returning, then, to the key question that was raised at the start of this review, i.e., whether roughness can be experienced multisensorially or whether instead it is only ever experienced within individual senses and thus the term is used metaphorically. The literature reviewed here suggests that roughness can be experienced crossmodally, at least by vision and touch. This, in turn, supports the amodal nature of roughness perception, at least if defined in terms of the pick up by two or more senses (i.e., vision and touch). Such a conclusion is suggested also by the existence of consistent crossmodal correspondences involving roughness, at least across specific sensory domains and regarding specific features (auditory roughness-visual shapes, see Giannos et al., 2021; Liew et al., 2018; Liew et al., 2017; hue-tactile roughness, see Ludwig & Simner, 2013; Slobodenyuk et al., 2015; see Table 2). It is perhaps also worth noting here that the seemingly natural use of the term roughness across a diverse range of experiences, and its seemingly easy interpretation by those who come across the term being used outside of a purely auditory/tactile context, might be taken to support that the term identifies an amodal or intermodal concept.

**Table 2:** Summary of the documented crossmodal correspondences involving roughness

Sensory domain	Main findings and relevant literature
Audiovisual	Auditory dissonance correlates with visual roughness and spikier images (Giannos et al., 2021; Liew et al., 2017; Liew et al., 2018). Dark/light colours associated with rough/less rough sounds (Sun et al., 2018).
Audiotactile	Low pitch matched with rough textures and high pitch with softness and with smooth textures (Eitan & Timmers, 2010; Etzi et al., 2016; Hamilton-Fletcher et al., 2018). Roughness tends to be associated with minor tonality (Murari et al., 2015). High vs. low music softness enhances consumers’ haptic softness perception (Imschloss & Kuehnl, 2019).
Visuotactile	Softness and smoothness matched to bright colours (i.e., yellow, pink, and white), while roughness matched to darker colours (i.e., black, brown, red and purple-red; Jraissati et al., 2016; Ludwig & Simner, 2013; Slobodenyuk et al., 2015).
Touch & Taste	Roughness associated with saltiness (Van Rompay & Groothedde, 2019; though see Wan et al., 2014). Biscuits tasted from roughers containers were rated as crunchier than those tasted from smooth containers (Biggs et al., 2016; Piqueras-Fiszman & Spence, 2012). Wine is judged to be significantly fruitier, sweeter, and more pleasant when tasters simultaneously touched smooth fabrics (Wang & Spence, 2018). Mineral water perceived as fresher, more pleasant, and lighter when contained in cups that felt smoother (Risso et al., 2019). Coffee tastes sweeter when sampled from a cup with a smooth, as opposed to a rough, surface. Coffee also rated as more acidic from the rough cup than when tasted from the smooth cup (Carvalho et al., 2020).
Touch & Olfaction	Fabric swatches judged as feeling softer in the presence of a pleasant odor (i.e., lemon or lavender) than in the presence of an unpleasant animal-like odour (Demattè et al., 2006, though see Koijck et al., 2015).
Auditory & Taste	Bitter taste mapped to rough and low-pitched sounds, whereas sweet tastes mapped to high pitched and smooth sounds (Knöferle et al., 2015). Saltiness associated with high auditory roughness (Wang et al., 2021).

Roughness can thus be added to the list of dimensions of perceptual experience that have been considered by experimental psychologists as amodal, intermodal, intersensory or, simply, universal, such as brightness, intensity, duration, shape (Lewkowicz & Turkewitz, 1980; Smith, 1994; Spence & Di Stefano, 2022b; von Hornbostel, 1931, 1950). Thus, on the one hand, Aristotle was apparently right when he included roughness among the “common sensibles.” However, on the other hand, the existence of common sensibles does not necessarily imply, as Aristotle seemingly suggested, the existence of the *sensus communis* (i.e., a common sense responsible for monitoring and coordinating the five senses out of which unified conscious experience arises). Indeed, Aristotle posited the *sensus communis* as a peculiar sixth sense, lacking a dedicated sensory apparatus. By contrast, the literature that has been reviewed here advocates that it is by no means necessary to admit the *sensus communis* to account for intersensory qualities. In fact, assuming that different modalities can provide independent information concerning roughness, there is no need to integrate such information since focusing on a single sense modality is typically sufficient to obtain all available data.

Another key question we aimed at elucidating regarded the use/meanings of the term *roughness* in the unisensory context. On the basis of the literature reviewed here, it would appear that people use the word *roughness* to refer to (at least) two different phenomena, namely, a primarily temporal and auditory one, and a primarily spatiotemporal and tactile one. As a temporally perceived stimulus dimension, roughness essentially characterizes auditory musical (i.e., dissonances) and nonmusical stimuli (i.e., speech, human and animal vocalizations, environmental sounds). As a spatiotemporal stimulus dimension, roughness can be attributed to visual, tactile and, to some extent, oral-somatosensory stimuli. These different properties elicit (at least partially) different responses in the perceiver. The auditory notion of roughness is typically perceived as unpleasant, triggering attentional behaviours and eliciting aversive reactions in the listener. Spatiotemporal roughness might be perceived as unpleasant, although it is to a certain extent appreciated in some specific contexts (e.g., wine tasting). Results showing that spatial rather than merely temporal mechanisms are crucial for the neural coding of texture might provide further support for our claim (Connor & Johnson, 1992).

An additional aspect might further distinguish the two notions of roughness. In the auditory domain, the perceiver cannot clearly identify or distinguish the elements giving rise to roughness. By contrast, in the case of “tactile” roughness, even when visually experienced, the perceiver can, at least to some extent, identify the elements that contribute to determine surface roughness. This might recall Leibniz’s theoretical differentiation between confused/distinct “ideas,” a concept that, in Leibnizian terminology, also includes perceptions (Leibniz, 1989). According to Leibniz, an idea is distinct when one can catalogue all the marks, or criteria, distinguishing that idea from others. Leibniz believed that most sensory ideas, such as those of color, are confused, because, though we reliably distinguish blue from red, we cannot spell out the marks or causes which make one object blue and another red. Thus, in Leibnizian terms, auditory roughness can be conceived of as a confused idea, while visuotactile can be considered as a clear one. Such a phenomenological distinction might be due to the different sensibility/discrimination thresholds of each sensory systems, and to the temporal versus spatiotemporal processing, meaning that auditory temporal resolution/information might not be sufficient to produce the distinct percept of roughness. This hypothesis is supported by the physiological and computational data reported by Gallace et al. (2012), according to which audition, compared with vision and touch, has a lower number of sensors and afferents, lower processing capacity in terms of bit/s, and lower portion of neocortex devoted to the processing of stimuli, thus making it outperformed by vision and touch in several perceptual/discriminatory tasks.

A final consideration concerns the experience of roughness in the chemical senses. The literature shows that, amongst different mouthfeel characteristics, astringency is associated with a “roughing” sensation. Astringency can be rightly considered a tactile sensation, as haptic roughness, resulting from the interaction between moving dermal surfaces, such as tongue–palate and tongue–food interactions (Chen & Stokes, 2012). However, an astringent mouthfeel has been linked to the roughness of the receptor surface rather than the surface being felt. Hence, it might be meaningful that people use a different term for mouthfeel and surface texture (i.e., astringency and roughness, respectively,^19^ perhaps denoting in this way a different perceived quality). And, probably, none would use the term *astringency* to describe visual shapes, fabrics, or sounds.

## Pitfalls of the reviewed literature: Theoretical and methodological issues

As has become evident from this review, roughness perception has been thoroughly investigated over recent decades using a wide variety of behavioural and neurophysiological protocols, ranging from simple tactile discrimination tasks to the recordings of brain activations during multisensory perception. Hereinafter, we highlight some limitations of the reviewed literature and, in the section Future Research Questions and Methodologies in the Study of Roughness Perception, we outline a number of key directions for future research in the area of multisensory roughness perception.

First, despite the impressive abundance and relative consistency of the accumulated evidence, the effort put in trying to replicate and strengthen previous evidence has been apparently scarce (with some exceptions, such as the works by Lederman). However, the knowledge on roughness perception would surely benefit from replication studies with larger samples and refined experimental protocols that might shed further light on those issues that were not completely explained by previous research (e.g., the role of spatial and temporal processing in the perception of fine surface texture). Relatedly, while several cross-cultural protocols have been carried out to investigate the unimodal perception of roughness (especially in audition, e.g., McDermott et al., 2016), cross-cultural approaches to the study of crossmodal perception of roughness are still scarce (see, e.g., Wan et al., 2014), thus leaving open the question about the universality of the observed crossmodal matchings (cf. Henrich et al., 2010).

Second, and more specifically, one might be impressed by the disproportionate amount of research conducted with sandpaper, which is a stimulus that participants rarely feel in their daily life. While using ad hoc stimuli is straightforward when the investigation deals with perceptual stimuli that are normally absent (or very rarely perceived) in human perceptual environment (e.g., musical chords or timbres), for haptic roughness it would seem far more natural to pick out stimuli from our daily environment. For instance, fabrics and natural elements (e.g., wood, stones, leaves) represent a potentially infinite sample of materials to choose from, allowing to expose participants to more ecologically salient stimuli. Moreover, studies rarely allow naturalistic tactile exploration, rather giving rigid constraints for the sake of methodological rigour. Given the above concerns, one could also observe that less frequently experienced stimuli, such as sandpaper, might capture participants’ attention more than the more frequently experienced ones, such as fabrics, thus ensuring that the experimental tasks are attentively carried out by participants.

Finally, as far as regards the experimental design, very few studies have investigated trimodal interactions (e.g., audio-visual-tactile, and/or smell; e.g., Speed et al., 2021), though these are allegedly the way we normally experience perceptual objects in our everyday life.

## Future research questions and methodologies in the study of roughness perception

While the term *roughness* is relevant in studies on audition, vision, touch, and taste, it apparently plays only a marginal role in the characterization of odours.^20^ In one of the very few studies that tangentially focus on roughness in (orthonasal) olfaction, Yoshida (1964) used different methods (i.e., multidimensional scaling, semantic differential scales) to investigate basic criteria that people used to classify their smelling experiences. Roughness (as opposed to smoothness) was included in the list of adjectival opposite that might be used to describe odours, together with adjectival pairs such as pleasant–unpleasant, heavy–light, sultry–refreshing, dirty–neat, dark–bright. Multidimensional scaling and semantic differential scales led to the extraction of different series of factors such as resinous, burnt, sweetness, and sensory pleasure, harshness, and vividness, respectively. Although roughness affects harshness, the latter is referred to as a wider factor, opposed to mildness and mostly affected by qualities such as delicateness, definition, softness. In the future, therefore, it will be interesting to provide additional evidence to determine whether and how roughness affects (orthonasal) olfaction in the unisensory context, as well as investigating the role of roughness in crossmodal associations or interactions involving olfaction, which have been much less investigated with respect to other crossmodal combinations (e.g., audiovisual, audiotactile, auditory/touch gustation). The recent study by Speed et al. (2021) went in this latter direction, investigating the crossmodal associations with auditory, olfactory, and tactile stimuli in children and adults. Finding little evidence for crossmodal associations in young children, the study suggests that experience plays a crucial role in crossmodal associations from sound, touch, and smell to other senses, thus opening to the question of which factor(s) prompt(s) the association (e.g., statistical learning, language, emotional mediation).

Another allegedly underinvestigated question in the multisensory research is how the graininess of foods influences the perception of visual stimuli that are experienced while eating. In this regard, it might be worth mentioning here the *Tate Sensorium*, a pioneering exhibition that took place in 2015 in which four paintings were deliberately matched with sounds, odours, tastes, and visual stimuli (Pursey & Lomas, 2018). For instance, Francis Bacon’s 1945 painting *Figure in a Landscape* was paired with a custom-made chocolate with a deliberately gritty texture (of cocoa nibs) that was supposed to intensify the harshness of the painting (Davis, 2015; Pursey & Lomas, 2018). Similar research might also shed light on a related fundamental question—that is, whether oral-somatosensory roughness is to be conceived of as a different kind of roughness perception, or simply as a different context in which the same (i.e., tactile) perceptual property is apprehended.

A further specific question that might deserve attention in the future regards the aesthetic effect of visual roughness. Literature in this regard is scarce and reported that visual roughness is not consistently rated as either aesthetically pleasant or unpleasant (Van Egmond et al., 2009).

Findings on the crossmodal influence between audition and touch might be exploited for marketing purposes. For example, retailers could potentially benefit from the music they choose to play in their environments to influence consumers’ perception of the merchandize. Given the importance attributed by consumers to touching the products before buying them in physical stores, and especially of softness as a highly salient feature (e.g., Workman, 2010), retailers might deliberately plan to choose the auditory stimuli that are known to modulate roughness perception. Results from Imschloss and Kuehnl (2019) confirmed that playing high rather than low music softness enhances consumers’ haptic softness perception, which, in turn, results in more positive product evaluations and willingness to pay. Findings from similar research might also inspire future marketing strategies for online channels (e.g., addressing how to compensate buyers for the absence of touch information; e.g., Silva et al., 2021).

As far as concerns research methodology, the study of roughness perception might benefit in the future from the use of several advanced technologies (see See et al., 2022, for a recent review). For example, electrovibration^21^ might be used to provide touch screen users of a tactile feedback, thus creating easy-to-use and easy-to-implement experimental setups for the investigation of crossmodal associations involving auditory, visual, and tactile stimuli. Similar technologies might also be exploited within non-research domains, such as mobile applications including communication, games, education, data visualization, in addition to devices for sensory substitution (e.g., for the blind).

Another line of research that will likely grow in the future is based on the use of virtual reality (VR). An example of this approach is the recent study by Günther et al. (2022), who presented a prototype for arm stroking and compared the effects of different visualizations on the perception of physical textures with distinct roughnesses. Similar approaches can potentially be used to exploit the programmable capabilities of virtual reality, so that the limit of aware recognition between surfaces with different roughness can be measured with more precision than in conventional methods (see also Drewing et al., 2004a; Gong et al., 2002; Hilsenrat & Reiner, 2010; Romano & Kuchenbecker, 2011). Furthermore, similar protocols can be developed using (haptic) mixed/augmented reality (e.g., Culbertson & Kuchenbecker, 2017), midair haptics (Beattie et al., 2020; Vi et al., 2017), as well as a combination of all technologies (Vaquero-Melchor & Bernardos, 2019). However, those technologies are not without their own limitations (see Rakkolainen et al., 2020, for midair haptics), which concern temporal and spatial precision of the stimulation, strength and range of stimulation, the bulkiness and safety of the system and, last but not least, its cost, especially when compared with the cost of traditional stimuli, such as sandpapers or fabrics.

## Conclusions

As this review of the literature has made clear, sensory roughness seems to be primarily experienced in certain domains (i.e., auditory and visuotactile), in which the term likely refers to two distinct perceptual properties: a temporally based property (i.e., auditory roughness) and a spatiotemporal one that is picked out by vision and touch and is related to surface texture. A tactile notion of roughness is also present in the oral-somatosensory (i.e., food science) literature, where it is typically referred to as astringency.

In audition, the literature has established a direct link between the roughness of auditory stimuli and aversion. In the natural world, rough auditory stimuli signal potential danger or harm, which are likely to be perceived as unpleasant. In a visuo-tactile context, roughness is also typically perceived as less pleasant than smoothness, although apparently not necessarily as being related to any natural/biological threat, and likely reflecting the combination of sensory and aesthetic responses. Finally, in the domain of gustation, roughness is a defining factor of astringency, a mouthfeel characteristic that provides a key contribution to our experience of several foods and beverages, from dark chocolate to coffee and wine.

As far as concerns roughness across the senses, a number of crossmodal interactions or associations have been demonstrated in the literature (see Table 2). Studies provided evidence that auditory roughness affects the perception of tactile roughness in the context of multisensory integration and suggested that tactile roughness is crossmodally associated to low pitch sounds and, at least to some extent, to minor mode. Haptic softness and smoothness tend to be matched to bright colours (i.e., yellow, pink, and white), while roughness to darker colours (i.e., black, brown, red, and purple-red). In those tasks that have assessed visuotactile associations, roughness has typically been matched with dark colours. Focusing on different visual features, or objects, such as shape, studies have demonstrated that auditory dissonance, either presented as isolated intervals or harmonized melodies, is highly correlated with visual roughness (and vice versa). Consistent crossmodal associations have been also demonstrated in the domain of taste and gustation, with roughness typically being associated with saltiness.

In order to fill the gap in the existing literature and to advance our understanding of multisensory roughness perception, future research might take advantage of technological devices (e.g., augmented/virtual reality, mid-air haptics) that allow for the investigation of roughness perception in, and across, different senses, i.e., vision, touch, audition. These technological settings might be implemented with devices for delivering olfactory stimuli, so as to make participants’ experience of roughness as sensorially rich as it typically is during ecological perception. The evidence gathered in such a multisensory experimental environment might eventually provide further precious insights to answer the Aristotelian question we start our review with: Is roughness, in the end, an amodal property?

Open practice statement

The review does not present any original results and, hence, no original experiment was preregistered.

## Footnotes

1 Although oral somatosensation might have an important role as well, see section Food Texture, Astringency, and (Oral-Somatosensory) Roughness.

2 Zwicker and Fastl (2006) defined the roughness of one asper (a Latin word for *rough*), the effect of a 60-dB, 1-kHz tone that is 100% modulated in amplitude at a modulation frequency of 70 Hz.

3 A vibrating membrane will produce harmonics as the result of increasing nonlinearity as the frequency (membrane tension) falls (Greenewalt, 1968). Low-frequency sounds will automatically be accompanied by increased harshness due to harmonic production and off tonal sounds produced by a flaccid vibrating membrane.

4 Pitch has also been associated with size, with high-pitched sounds matched with small objects and low-pitched sounds being matched with large objects (e.g., Evans & Treisman, 2010; Gallace & Spence, 2006; Parise & Spence, 2009; Parise & Spence, 2012; though see Antovic, 2009; and Eitan et al., 2014, for different associations in a dynamic context).

5 By contrast, a recent study by Postal et al. (2020) revealed that laboratory mice (C57Bl6J) have a preference for rougher auditory stimuli. However, in contrast with other evidence from rats (e.g., Crespo-Bojorque & Toro, 2016; Fannin & Braud, 1971), C57Bl6J mice were unable to discriminate between consonant and dissonant stimuli, thus suggesting that further research is needed to better assess auditory perception in those mice.

6 The auditory effect produced by the object also depends on the spatial distribution, size, and shape of surface elements, and, in particular, on the distinction between the macro- and micro-geometric elements (see section Surface Roughness: Touch and Vision).

7 The research shows that the visual system also explores external information in an active, dynamic way by means of a series of eye movements and fixations over the stimulus of interest (see Findlay & Gilchrist, 2003, for a review). However, a study comparing exploration strategies across vision and touch for texture perception, suggests that these strategies appear to be modality specific (Sailer et al., 2003).

8 However, other studies have suggested that, depending on the shapes of the surface elements, the relationship between perceived roughness and spacing might result in an inverted U-shape (e.g., see Blake et al., 1997; Drewing, 2016).

9 Given the distinct processing mechanisms of visual and tactile roughness, the results from Lederman and Abbot (1981) might seem surprising. One might be tempted to put forward the existence of some kind of predictive coding account according to which, when texture is experienced visually, our brain imagines/predicts the tactile effect of that specific texture. In such a case, visual prediction and haptic reality would naturally align (see Sun et al., 2016, for relevant neurophysiological findings; see also Viengkham et al., 2019).

10 This might induce one to speculate about the similar acuity for roughness of the two senses. However, a direct comparison of the discriminatory thresholds goes far beyond the scope of this review. Moreover, comparing acuity in dynamic explorative tasks is a complex issue, as the combination of several factors contribute to determine the sensory thresholds, such as the dimensions and density of surface elements for spatial acuity, distance, and angle of observation for visual acuity. In addition to those elements, individual/subjective factors might also have an effect on discriminatory abilities, not to mention the active versus passive nature of touch, and the particular skin surface being stimulated.

11 A similar observation could be put forward for tactile roughness coding on the fingertip (e.g., Weber et al., 2013).

12 ‘Roughness’ ranked fourth out of 15 in the most used terms, being mentioned in 38% of the participants descriptions (though the first two most used terms are very general—that is, *sensation* and *mouth*, and the third is *dry*, which is also related to roughness).

13 One might also note that the tactile information we get from exploration of a surface with the tongue is much richer than that obtained via exploratory touch involving the finger or hand. Thus, licking might be useful to detect tactile differences that perhaps appear to the eye but which cannot be felt by hand. However, the use of tongue to gather tactile information about surface roughness is much less frequent than its use to gather data concerning edible foods. The above distinction between the quality of the information that it is possible to get from tongue vs. finger exploration would probably rely on different contact mechanics, given that both the oral cavity and palmar surface of the hand are similar in terms of innervation density (16-19.000 fibers and 17.000, respectively; see Corniani & Saal, 2020).

14 “The senses’ intimate link—the existence of intersensory qualities like brightness, intensity, roughness, etc.—all of this is based on the fact that the psycho-physical organism reacts as a whole, before any separation into distinct spheres of sensitivity” (our translation).

15 Interestingly, such crossmodal correspondence might be exploited for marketing purposes (see e.g., Imschloss & Kuehnl, 2019; we briefly expand on this line of research in the Section Future Research Questions and Methodologies in the Study of Roughness Perception).

16 Interestingly, however, pitch and hue can also both be represented as circular dimensions. In fact, according to Pridmore (1992), they are the only perceptual dimensions that can be so represented (see also Spence & Di Stefano, 2022c on this theme).

17 Van Rompay and Groothedde (2019) have reported that in the tactile domain saltiness is associated with textural irregularity and roughness, which obviously mimic the texture of salt crystals themselves (as well as sugar crystals).

18 https://www.sfgate.com/cooking/article/A-new-old-restaurant-trend-eating-with-your-5551364.php.

19 The two qualities are distinguished in languages other than English, such as Italian (“astringenza”), French (“astringence”), Spanish (“astringencia”), German (“Adstringenz”). Each term derives from the Latin verb “adstringo,” that means “to restrict” or “to bind.”

20 Intriguingly, early Greek atomists suggested that the atoms of sweet and pleasant-smelling substances had smooth surfaces, whereas atoms of acidic materials had spiky shapes that pricked and irritated the nose (Sell, 2006).

21 Electrovibration is a technique that enables the generation of haptic effects on a screen by applying an alternating voltage to the conductive layer of a surface capacitive touch screen. By controlling the amplitude, frequency, and waveform of the input voltage, the frictional force between the moving finger and the touch screen can be modulated (see Vardar et al., 2017).
